# Differential requirement for dimerization partner DP between E2F-dependent activation of tumor suppressor and growth-related genes

**DOI:** 10.1038/s41598-018-26860-0

**Published:** 2018-05-31

**Authors:** Hideyuki Komori, Yasuko Goto, Kenta Kurayoshi, Eiko Ozono, Ritsuko Iwanaga, Andrew P. Bradford, Keigo Araki, Kiyoshi Ohtani

**Affiliations:** 1 Life Sciences Institute, University of Michigan, 210 Washtenaw Avenue, Ann Arbor, MI 48109-2216 USA; 2 Department of Biomedical Chemistry, School of Science and Technology, Kwansei Gakuin University, 2-1 Gakuen, Sanda, Hyogo, 669-1337 Japan; 3 Chromosome Replication Lab, The Francis Crick Institute, Midland Road, London, NW1 1AT UK; 4 Department of Craniofacial Biology, University of Colorado School of Dental Medicine, Anschutz Medical Campus, 12801 East 17th Avenue, Aurora, CO 80045 USA; 5 Department of Obstetrics and Gynecology, University of Colorado School of Medicine, Anschutz Medical Campus, 2800 East 19th Avenue, Aurora, CO 80045 USA

## Abstract

The transcription factor E2F plays crucial roles in cell proliferation and tumor suppression by activating growth-related genes and pro-apoptotic tumor suppressor genes, respectively. It is generally accepted that E2F binds to target sequences with its heterodimeric partner DP. Here we show that, while knockdown of DP1 expression inhibited ectopic E2F1- or adenovirus E1a-induced expression of the *CDC6* gene and cell proliferation, knockdown of DP1 and DP2 expression did not affect ectopic E2F1- or E1a-induced expression of the tumor suppressor *ARF* gene, an upstream activator of the tumor suppressor p53, activation of p53 or apoptosis. These observations suggest that growth related and pro-apoptotic E2F targets are regulated by distinct molecular mechanisms and contradict the threshold model, which postulates that E2F activation of pro-apoptotic genes requires a higher total activity of activator E2Fs, above that necessary for E2F-dependent activation of growth-related genes.

## Introduction

E2F family proteins (E2F1-E2F5) form heterodimeric complexes with DP family proteins (DP1 and DP2), generating E2F transcriptional activity that regulates expression of growth-related target genes^1–3^. E2F activity is regulated by retinoblastoma (RB) family proteins (pRB, p107 and p130) during the cell cycle. RB family proteins directly bind to and inactivate E2F in the resting state. Normal growth signals, such as serum stimulation of fibroblasts, induce phosphorylation of RB family proteins by cyclin-dependent kinases (CDKs), resulting in an increase in E2F transcriptional activity, activation of the growth-related target genes, and subsequent cell cycle progression^1–3^. Since the DP subunit is an obligate partner for efficient DNA binding of E2F to canonical E2F response elements (TTT^C^/_G_^G^/_C_CGC) in growth-related target promoters, it is also essential for promotion of cell cycle progression^4,5^.

In addition to their role in cell cycle progression, E2F proteins, especially E2F1, also play a key role as mediators of apoptosis^1–3^. Ectopic expression of E2F1 or deregulated endogenous E2F activity resulting from expression of adenovirus E1a, which binds to and inactivates the RB family proteins, induces apoptosis in serum-starved fibroblasts^6–8^. Accordingly, ectopic expression of E2F1 or E1a activates pro-apoptotic E2F target genes, including the tumor suppressor *ARF* gene, an upstream activator of the tumor suppressor p53^1–3,8–10^, which results in activation of p53 and induction of apoptosis.

pRB plays a crucial role in tumor suppression mainly through restraining E2F activity. The *RB1* gene, which codes for pRB, is inactivated or mutated in many forms of human cancer, resulting in enhanced, or deregulated E2F activity, due to loss of pRB function^11,12^. Hereafter, we refer E2F activity induced by loss of pRB function or ectopic expression of E2F as deregulated E2F activity, and E2F activity induced by growth stimulation as physiological E2F activity. Upon loss of pRB function, the *ARF* gene is activated by deregulated E2F, resulting in activation of p53^9^. Mice heterozygous for *RB1* develop pituitary tumors^13^ and loss of *ARF* accelerates tumorigenesis^14^. In addition, tissue specific inactivation of *p53*, when combined with pRB inactivation, promotes the development of lung and ovarian tumors in mice^15,16^. These observations suggest that E2F regulation of the ARF-p53 pathway is crucial for prevention of tumorigenesis resulting from dysfunction of pRB.

We previously showed that E2F regulation of the *ARF* gene is distinct from that of growth-related target genes in that, unlike growth-related E2F targets, which are activated by both physiological and deregulated E2F activity, the *ARF* gene specifically responds to deregulated E2F activity^17^. In human normal fibroblasts, the ARF promoter responds to deregulated E2F activity induced by ectopic expression of E2F1 or inactivation of pRB by adenovirus E1a or short hairpin RNA (shRNA), but not to physiological E2F activity induced by serum stimulation. Growth-related target promoters, on the other hand, are activated by all of these stimuli. These observations suggest that the ability of the ARF promoter to discriminate between physiological and deregulated E2F activity serves as the basis for the *ARF* gene to function as a tumor suppressor gene in response to dysfunction of pRB^17^. To gain insight into the mechanisms, by which the ARF promoter is selectively activated by deregulated E2F activity, we identified and characterized the E2F-responsive element of ARF promoter (EREA). In contrast to typical E2F binding sequences (TTT^C^/_G_^G^/_C_CGC) in growth-related target promoters, EREA lacks the T nucleotides and is composed solely of GC repeats. ChIP assay showed that E2F1 bound to EREA upon over-expression of E2F1 or expression of E1a but not upon serum stimulation in normal human fibroblasts. These results suggest that EREA specifically binds deregulated E2F1.

The molecular basis of how E2F discriminates target genes involved in the opposite cell fates of cell proliferation and apoptosis is not known. To explain E2F regulation of both cell proliferation and apoptosis, the threshold model has been proposed^2^. In this model, a total pool of free activator E2Fs contributes to E2F transcriptional activity, which, upon reaching a critical threshold, induces growth-related genes that are involved in cell proliferation. Once E2F activity exceeds a second higher threshold, pro-apoptotic genes are activated, triggering cell death. However, given that the sequence of the atypical E2F binding site in the ARF promoter (EREA) is distinct from that of canonical E2F binding sites in growth-related target promoters, we postulated that E2F recognition of and interaction with EREA may be distinct from that of typical E2F binding sites, such that E2F binds to EREA by itself or together with a binding partner other than DP. We thus hypothesized that E2F regulation of the *ARF* gene may not conform to the threshold model or requirement for the heterodimeric partner DP.

In contrast to the established functions of DP in growth-related E2F-dependent gene expression and cell cycle progression, the role of DP in deregulated E2F-mediated pro-apoptotic gene expression and apoptosis has not been elucidated. Thus, in this study, we examined the requirement of deregulated E2F for DP in induction of *ARF* gene expression and apoptosis, in human normal fibroblasts, using shRNAs against *DP1* and *DP2* mRNA. The threshold model predicts that *ARF* gene expression and apoptosis would require higher E2F activity and therefore be more dependent on DP than E2F-dependent growth-related gene expression and cell cycle progression. Surprisingly, our results show that, while DP is essential for deregulated E2F induction of growth-related *CDC6* gene expression and cell cycle progression, activation of *ARF* gene expression and apoptosis is independent of DP. These results contradict the threshold model and suggest that E2F regulation of pro-apoptotic tumor suppressor genes is not simply a function of elevated total E2F activity in concert with DP family members.

## Results

### ShRNAs against DP1 mRNA transduced by adenovirus vector down regulate DP1 expression in human normal fibroblasts

**Figure 1:**
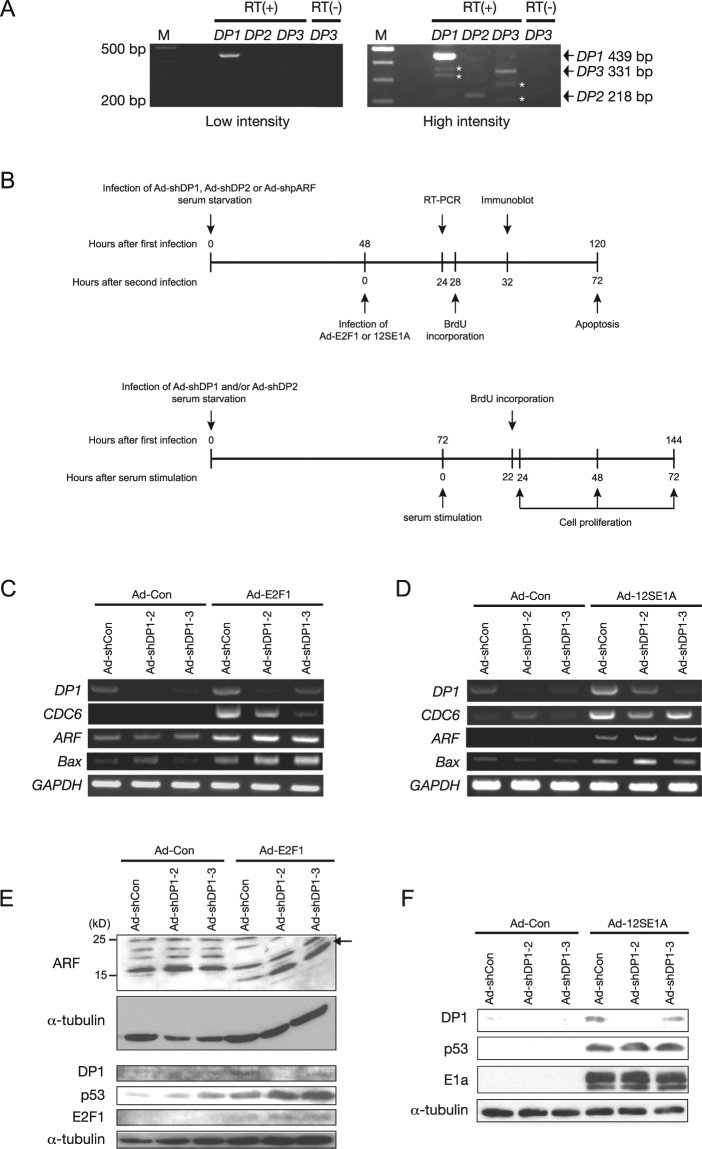
DP1 knockdown does not compromise E2F-induced *ARF* mRNA expression or activation of p53. (**A**) Expression of DP family members in human normal fibroblast WI-38 cells. RT-PCR analysis of *DP* family genes with 25 cycles of PCR using same amount of cDNA. *Non-specific bands. Full-length gel is presented in Supplementary Fig. S1. (**B**) Time frame of knockdown experiments. (**C**,**D**) RT-PCR analysis of mRNA induction by ectopic expression of E2F1 (**C**) or E1a (**D**) in WI-38 cells expressing *DP1*-shRNA. Full-length gels are presented in Supplementary Figs S2(C) and S3(D). (**E**,**F**) Immunoblot analysis of DP1 expression and induction of ARF and p53 by ectopic expression of E2F1 (**E**) or E1a (**F**) in WI-38 cells expressing *DP1*-shRNA. Full-length blots are presented in Supplementary Figs S4(E) and S5(F).

To determine the role of DP proteins in the induction of *ARF* gene expression and apoptosis by deregulated E2F, we established shRNA-mediated knockdown of DP1 expression in human normal fibroblasts. We focused on DP1 as it is the predominantly expressed DP family member in human normal fibroblasts (Fig. 1A)^5^, over DP2, which cooperates with E2F proteins in the same manner as DP1^1–3^. The third member of the DP family, *DP3*, is also expressed at low levels (Fig. 1A) and DP3 functions to inhibit E2F activity^18^. Furthermore, shRNA-mediated down regulation of DP1 alone, in human fibroblasts or the corresponding ortholog (dDP) in *Drosophila*, is sufficient to inhibit binding of E2F to typical E2F binding sequences, leading to a reduction in growth-related target gene expression and cell cycle progression^4,5^. These observations indicate that DP1 is the primary functional DP family protein in human normal fibroblasts, and thus a logical target to investigate the role of DP in E2F regulation of transcription, cell cycle progression and apoptosis.

We constructed recombinant adenoviruses expressing shRNA against four distinct regions of human *DP1* mRNA and found two of them (Ad-shDP1-2 and Ad-shDP1-3) were effective. As shown in Fig. 1B, normal human fibroblast WI-38 cells were infected with Ad-shDP1 and cultured for 48 h under serum-starved conditions. The cells were then infected with adenoviral E2F1 or E1a construct (Ad-E2F1 or Ad-12SE1A) and maintained under serum-starved conditions. Reverse transcription (RT)-PCR and immunoblot analyses revealed that the introduction of DP1 shRNAs successfully reduced the levels of *DP1* mRNA and protein in WI-38 cells ectopically expressing E2F1 or E1a (Fig. 1C–F). We also noted that ectopic E2F1 or E1a expression increased the levels of *DP1* mRNA and its protein (Fig. 1C–F), suggesting that the *TFDP1* gene may be a target of E2F. Under these conditions, expression of DP2 protein was not detected by immunoblot analysis even with ectopic expression of E2F1 or E1a (Supplementary Fig. S6).

### DP1 knockdown does not compromise induction of *ARF* gene expression or p53 by deregulated E2F in human normal fibroblasts

Using the DP1-knockdown system, we first examined the effects of DP1 knockdown on E2F regulation of target gene expression in WI-38 cells. As expected, knockdown of DP1 impaired production of *CDC6* mRNA induced by the ectopic expression of E2F1 or E1a (Fig. 1C and D). In contrast, ectopically expressed E2F1- or E1a-induced *ARF* mRNA and protein expression were not inhibited by DP1 knockdown (Fig. 1C–F). Indeed, a modest increase in E2F-stimulated *ARF* gene expression was observed. Consistent with the lack of effect on *ARF* gene expression, DP1 knockdown did not reduce the levels of p53 protein or *Bax* mRNA, a p53 target gene^19^, in WI-38 cells expressing E2F1 or E1a (Fig. 1C–F). These results indicate that, in contrast to the *CDC6* gene, deregulated E2F activation of the *ARF* gene is largely independent of DP1.

**Figure 2:**
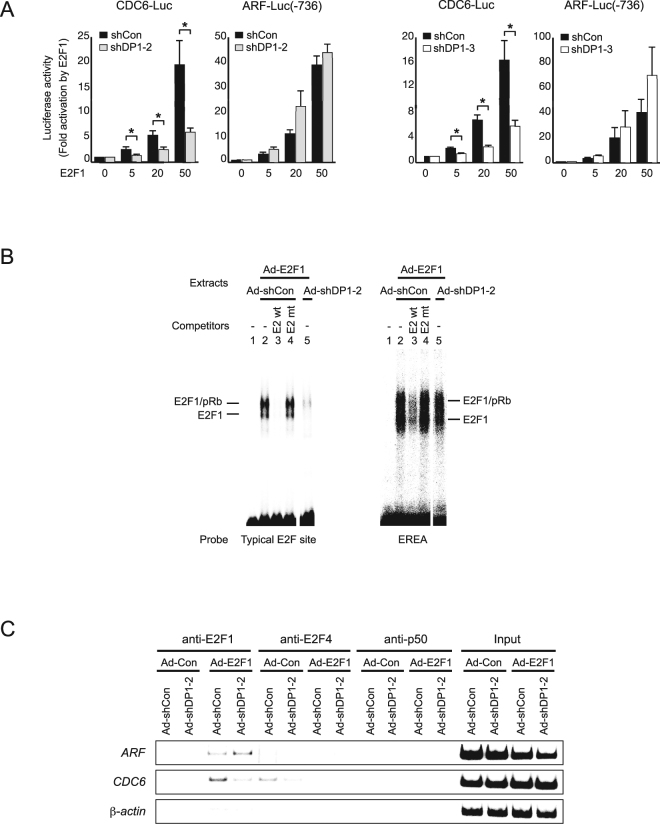
DP1 knockdown does not reduce E2F1 activation of the ARF promoter or binding of deregulated E2F1. (**A**) Reporter analysis of CDC6 and ARF promoters by ectopic expression of E2F1 in WI-38 cells expressing *DP1*-shRNA. *P < 0.05. (**B**) Gel mobility shift assays with extracts from WI-38 cells ectopically expressing E2F1 and *DP1*-shRNA. Probes were a DHFR promoter fragment containing typical E2F sites (left panel) and EREA (right panel). Competitors were an adenovirus E2 enhancer fragment containing typical E2F sites (E2 wt) and its mutant (E2 mt). Ad-shCon and Ad-shDP1-2 data were on the same gels with the same exposure (Supplementary Fig. S7). (**C**) ChIP analysis of E2F target promoters in WI-38 cells ectopically expressing E2F1 and *DP1*-shRNA. Full-length gels are presented in Supplementary Fig. S8.

To explore the molecular mechanism underlying the differential dependency on DP1 for induction of *CDC6* and *ARF* gene expression by E2F, we examined the functional role of DP1 in activation of the CDC6 and ARF promoters by deregulated E2F1 in WI-38 cells. Both promoters were activated by ectopically expressed E2F1 in a dose-dependent manner, with the ARF promoter being more sensitive than CDC6 promoter (Fig. 2A). DP1 knockdown significantly decreased E2F1-dependent activation of the CDC6 promoter (Fig. 2A). In contrast, knockdown of DP1 did not decrease dose dependent E2F1 activation of the ARF promoter (Fig. 2A). These results suggest that the differential requirement for DP1 between induction of *CDC6* and *ARF* gene expression by E2F1 is mediated by distinct elements in their respective proximal promoters. The CDC6 promoter contains canonical E2F binding sequences (TTTGGCG^G^/_C_)^20–22^. In contrast, the E2F-responsive element of the ARF promoter (EREA), necessary for activation of the *ARF* gene by deregulated E2F in human fibroblasts, is an atypical site composed of only GC repeats^17^. We therefore examined the effects of DP1 knockdown on the DNA binding activity of deregulated E2F1 to these distinct E2F response elements. We first performed gel mobility shift assays using typical E2F sites from DHFR promoter and EREA probes. Consistent with previous reports^4,5^, DP1 knockdown in WI-38 cells dramatically reduced the level of ectopically expressed E2F1 bound to the canonical DHFR E2F sites (Fig. 2B left panel). In contrast, DP1 knockdown did not significantly affect the level of ectopically expressed E2F1 bound to the atypical EREA (Fig. 2B right panel). We next examined the effect of DP1 knockdown on the DNA binding activity of ectopically expressed E2F1 *in vivo* by ChIP assay. While DP1 knockdown significantly reduced the level of endogenous E2F4 and ectopic E2F1 bound to endogenous CDC6 promoter, it did not reduce the level of ectopic E2F1 bound to endogenous ARF promoter (Fig. 2C). Rather a slight increase was observed. These results indicate that the differential effects of DP1 on the induction of endogenous *CDC6* and *ARF* gene expression by E2F1 is due to DP1-dependent binding of deregulated E2F1 to canonical E2F response elements of *CDC6* versus DP1-independent interaction of E2F with atypical E2F response element of *ARF*, respectively.

### DP1 does not cooperate with E2F1 to activate ARF promoter in human normal fibroblasts

**Figure 3:**
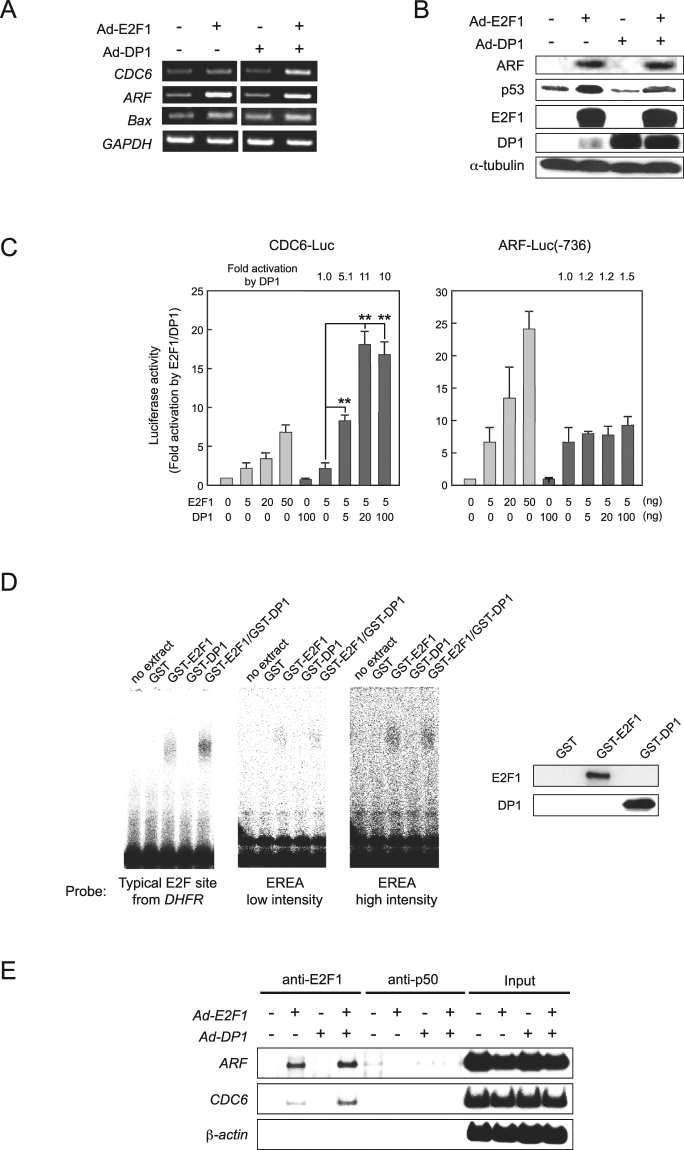
DP1 does not cooperate with deregulated E2F1 to bind to or activate ARF promoter. (**A**,**B**) RT-PCR analysis (**A**) and immunoblot analysis (**B**) in WI-38 cells infected with Ad-E2F1 (MOI 20) and/or Ad-DP1 (MOI 100). Full-length gels and blots are presented in Supplementary Figs S9(A) and S10(B). (**C**) Reporter analyses of CDC6 and ARF promoters in WI-38 cells expressing E2F1 and DP1. **P < 0.01. (**D**) Gel mobility shift assays using GST-E2F1 and GST-DP1. Probes were a DHFR promoter fragment containing typical E2F sites (left panel) and EREA (two middle panels). Western blot analysis of purified GST-E2F1 and GST-DP1 (right panel). Full-length blots are presented in Supplementary Fig. S11. (**E**) ChIP analysis of E2F target promoters in WI-38 cells infected with Ad-E2F1 (MOI 20) and/or Ad-DP1 (MOI 100). Full-length gels are presented in Supplementary Fig. S12.

DP1 is known to cooperate with E2F1 in binding and activation of typical E2F binding sequences^1,3,23^. To further investigate the role of DP1 in E2F-dependent regulation of the *CDC6* and *ARF* genes, we examined the effects of overexpression of DP1. Multiple lines of evidence indicate that, while DP1 enhances deregulated E2F induction of the *CDC6* gene, DP1 does not cooperate with deregulated E2F to activate the *ARF* gene in human normal fibroblasts. Firstly, co-expression of DP1 enhanced E2F1-induced *CDC6* gene expression, but did not enhance ectopic E2F1-induced *ARF* gene or protein expression (Fig. 3A and B). Consistent with this finding, ectopic DP1 had no significant effect on the level of E2F1-induced p53 protein or *Bax* mRNA (Fig. 3A and B). Secondly, co-expression of DP1 led to a strong increase in activation of the CDC6 promoter by the ectopic expression of E2F1 in WI-38 cells (Fig. 3C). In contrast, co-expression of DP1 did not significantly influence ectopic E2F1-mediated activation of the ARF promoter, although E2F1-mediated activation of the ARF promoter could be further enhanced by increased E2F1 expression (Fig. 3C). Thirdly, in gel mobility shift assays using purified GST-E2F1 and GST-DP1, GST-E2F1 alone was sufficient to interact with EREA under standard gel shift conditions (Fig. 3D, two middle panels). Notably, this interaction was not increased by addition of GST-DP1, suggesting that the E2F1/DP1 heterodimer has less affinity for EREA than E2F1 alone. In contrast, interaction of GST-E2F1 with a typical E2F site from DHFR promoter was significantly increased by addition of GST-DP1 (Fig. 3D, left panel), indicating that binding of an E2F1/DP1 heterodimer to typical E2F site is more efficient than E2F1 alone as previously reported^23^. Finally, consistent with the gel shift results, ChIP assay showed that, whereas ectopic DP1 dramatically increased binding of ectopically expressed E2F1 to the endogenous CDC6 promoter, it did not significantly impact the amount of E2F1 bound to the endogenous ARF promoter in WI-38 cells (Fig. 3E). These results indicate that DP1 does not cooperate with deregulated E2F1 to bind to EREA and to induce *ARF* gene expression in human normal fibroblasts.

### All DP family members do not contribute to E2F1-mediated induction of *ARF* gene expression

**Figure 4:**
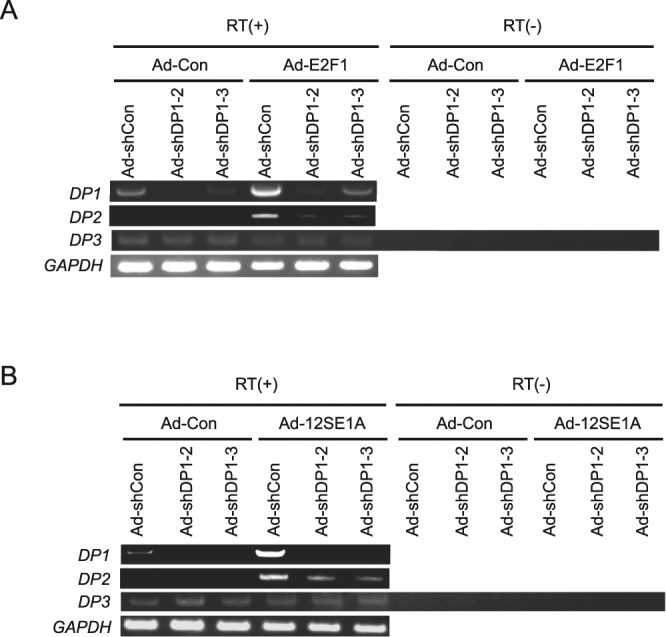
No compensatory increase in DP2 or DP3 and induction of DP1 and DP2 expression by ectopic expression of E2F1 or E1a in DP1-knocked down cells. (**A**,**B**) RT-PCR analysis of *DP* family members in WI-38 cells expressing *DP1*-shRNA and E2F1 (**A**) or E1a (**B**). Full-length gels are presented in Supplementary Figs S13(A) and S14(B).

**Figure 5:**
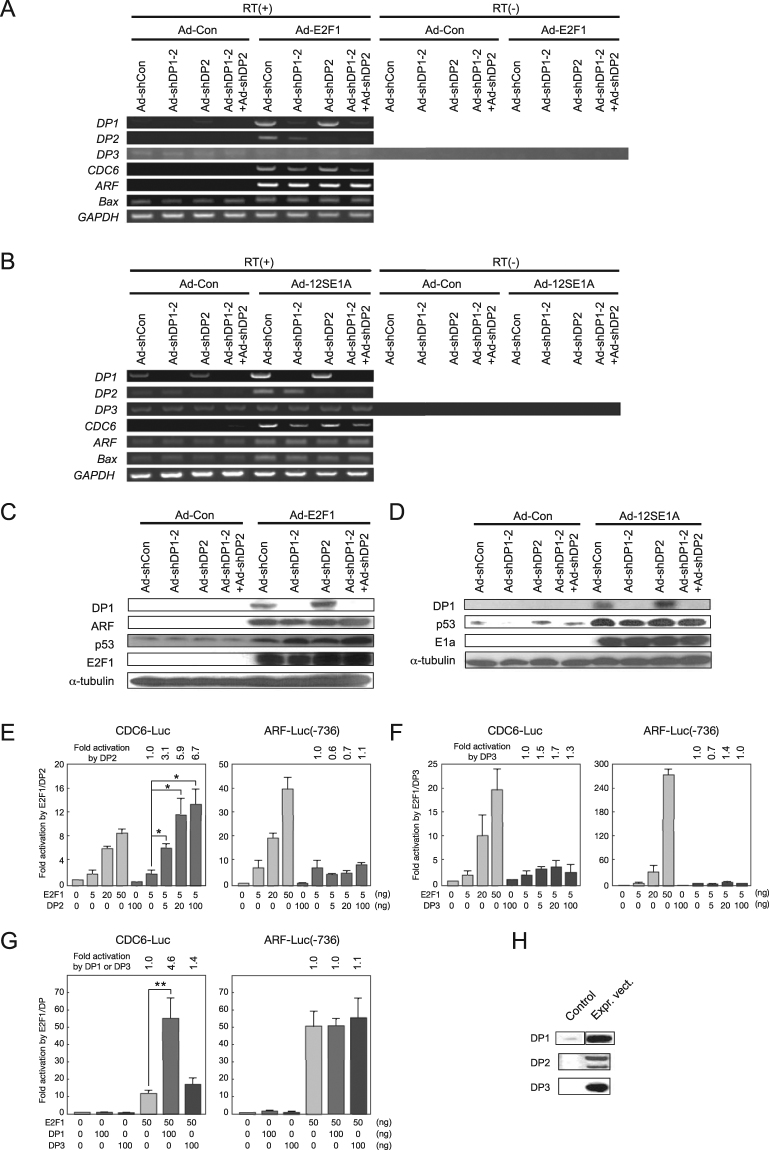
DP family proteins do not contribute to deregulated E2F-induced *ARF* mRNA expression or activation of p53. (**A**,**B**) RT-PCR analysis of mRNA induction by ectopic expression of E2F1 (**A**) or E1a (**B**) in WI-38 cells expressing *DP1*-shRNA. Full-length gels are presented in Supplementary Figs S15(A) and S16(B). (**C**,**D**) Immunoblot analysis of DP1 expression and induction of ARF and p53 by ectopic expression of E2F1 (**C**) or E1a (**D**) in WI-38 cells expressing *DP1*-shRNA. Full-length blots are presented in Supplementary Figs S17(C) and S18(D). (**E**–**G**) Reporter analyses in WI-38 cells ectopically expressing E2F1 and DP2 (**E**) or DP3 (**F**,**G**). *P < 0.05, **P < 0.01. (**H**) Immunoblot analysis of DP family proteins in 293 cells transfected with expression plasmid for respective DP family proteins. Full-length blots are presented in Supplementary Fig. S19.

We next examined the possibility that other DP family members may compensate for the effect of DP1 knockdown in E2F-mediated *ARF* gene expression. To address this issue, we examined the contribution of DP2 and DP3 to deregulated E2F activity, which induces *ARF* gene expression in WI-38 cells. Levels of *DP2* and *DP3* mRNAs were not increased upon DP1 shRNA expression, indicating that there was no compensatory increase in *DP2* or *DP3* gene expression in response to DP1 knockdown (Fig. 4A and B). However, the level of *DP2* mRNA was increased by ectopic expression of E2F1 or E1a, suggesting that the *DP2* gene may also be an E2F target. To examine whether DP2 may compensate for DP1 knockdown, we generated recombinant adenovirus expressing shRNA against human *DP2* mRNA to knock down DP2 expression. Introduction of DP2 shRNA clearly reduced *DP2* mRNA expression in WI-38 cells (Fig. 5A and B). However, unlike DP1, knockdown of DP2 did not reduce induction of *CDC6* mRNA expression by ectopic E2F1 or E1a. This result suggests that, in WI-38 cells, endogenous DP2 is not required for E2F-dependent gene expression. Consistent with this, combinatorial expression of DP2 shRNA and DP1 shRNA had no significant effect on deregulated E2F-induced expression of the *ARF* gene, p53 protein or the *Bax* gene in WI-38 cells (Fig. 5A–D). Furthermore, reporter analysis showed that co-expression of DP2 did not affect activation of the ARF promoter by ectopic E2F1 in WI-38 cells (Fig. 5E). In contrast, DP2 enhanced activation of the CDC6 promoter, in a similar manner to DP1. This result indicates that neither DP1 nor DP2 cooperate with E2F1 to activate the ARF promoter.

Co-expression of DP3 had no significant effects on E2F1 activation of the CDC6 or ARF promoter. DP3 has previously been reported to inhibit activation of E2F-responsive promoters by the ectopic E2F1 expression in the human liver cell line L02^18^. However, DP3 did not reduce ectopic E2F1-mediated activation of the CDC6 or ARF promoter in WI-38 cells, at varying amounts of E2F1 (Fig. 5F and G). We confirmed expression of DP3 protein as well as other DP family proteins from the DP expression vectors using 293 cells (Fig. 5H). Thus, unlike in L02 cells, DP3 may not play a significant role in regulating E2F activity in WI-38 cells. Collectively, these results indicate that all DP family members do not contribute to deregulated E2F-induced *ARF* gene expression in human normal fibroblasts.

### DP knockdown compromised cell cycle progression but not apoptosis induced by deregulated E2F in human normal fibroblasts

**Figure 6:**
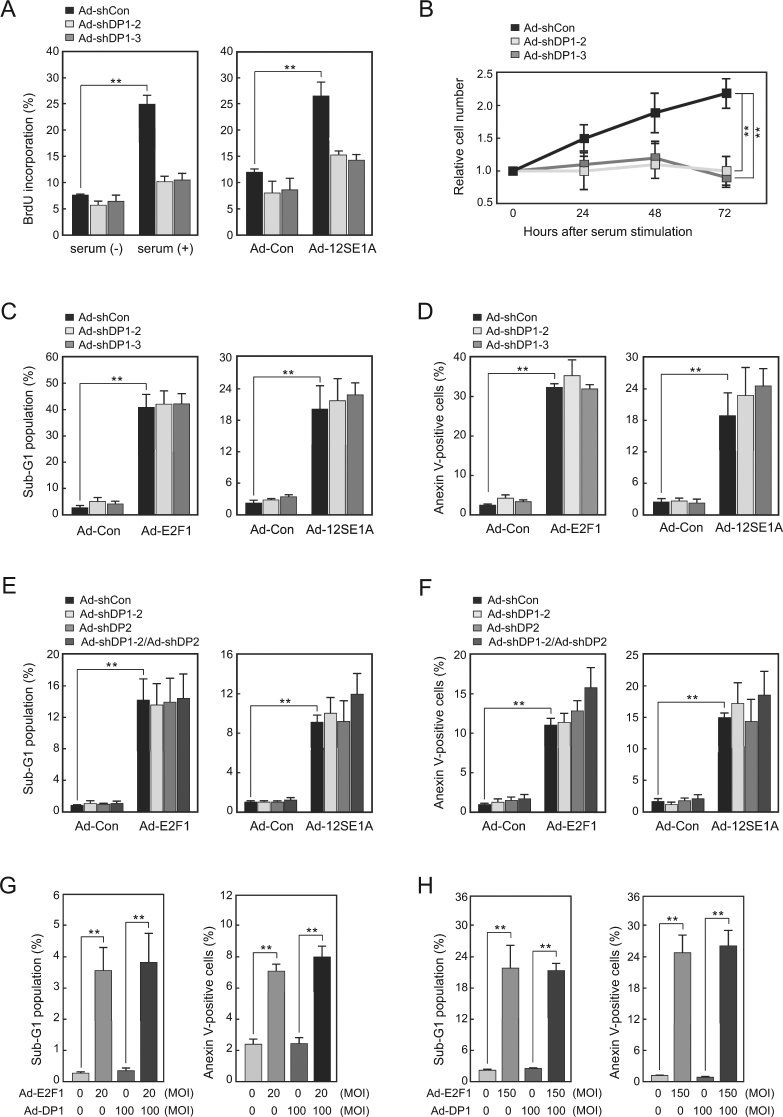
DP1 does not affect E2F-induced apoptosis. (**A**) DP1 knockdown suppresses DNA synthesis induced by serum stimulation or expression of E1a. FACS analysis of BrdU-positive cells in WI-38 cells expressing *DP1*-shRNA stimulated with serum or expressing E1a. **P < 0.01. (**B**) DP1 knockdown suppresses cell proliferation induced by serum stimulation. Growth curve of WI-38 cells expressing *DP1*-shRNA. **P < 0.01. (**C**–**F**) DP knockdown does not reduce deregulated E2F-induced apoptosis. (**C**,**D**) Percentage of cells with a sub-G_1_ DNA content (**C**) and annexin V-stained cells (**D**) in WI-38 cells infected with Ad-shDP1s and either Ad-E2F1 or Ad-12SE1A. **P < 0.01. (**E**,**F**) Percentage of cells with a sub-G_1_ DNA content (**E**) and annexin V-stained cells (**F**) in WI-38 cells infected with Ad-shDP1 and/or Ad-shDP2, and either Ad-E2F1 or Ad-12SE1A. **P < 0.01. (**G**,**H**) DP1 does not contribute to E2F1-induced apoptosis in WI-38 cells. Percentage of sub-G_1_ population (left panel) and annexin V-positive cells (right panel) in WI-38 cells expressing E2F1 and/or DP1. WI-38 cells were infected with Ad-E2F1 at low MOI (20) (**G**) or high MOI (150) (**H**). **P < 0.01.

DP1 knockdown reduced E2F-induced gene expression involved in cell cycle progression, but not E2F-induced *ARF* gene expression or p53 activity related to E2F-induced apoptosis. We therefore investigated the effect of DP1 depletion on E2F-mediated apoptosis. We first confirmed that knockdown of DP1 expression inhibits E2F-mediated cell cycle progression. WI-38 cells were transduced with DP1 shRNA by infection of Ad-shDP1s and rendered quiescent by serum-starvation. The cells were re-stimulated with serum at 72 h after the introduction of DP1 shRNA and harvested 22 h later to determine the percentage of cells incorporating BrdU or at indicated time points to determine the rate of proliferation (Fig. 1B). Both BrdU incorporation and the proliferation rate of WI-38 cells, re-stimulated with serum, were reduced to near basal levels by DP1 knockdown (Fig. 6A, left panel and 6B). DP1 knockdown also diminished BrdU incorporation into WI-38 cells expressing E1a (Fig. 6A, right panel). These results indicate that DP1 knockdown compromises physiological and deregulated E2F activity for promotion of cell cycle progression consistent with previous reports^4,5^.

To examine the effect of DP1 knockdown on E2F-mediated apoptosis, we measured the percentage of cells with a sub-G_1_ DNA content and that stained with annexin V by flow cytometry at 72 h after infection of WI-38 cells with Ad-E2F1 or Ad-12SE1A (Fig. 1B). Consistent with ectopic E2F1 or E1a mediated induction of *ARF* gene expression and activation of p53 being independent of DP1, DP1 knockdown did not reduce apoptosis induced by the ectopic expression of E2F1 or E1a (Fig. 6C and D). Similarly, combinatorial knockdown of DP1 and DP2 expression also had no significant effect on deregulated E2F-induced apoptosis in WI-38 cells (Fig. 6E and F). These results indicate that, in contrast to E2F mediated cell cycle progression, deregulated E2F induction of apoptosis is not dependent on DP in human normal fibroblasts. We also examined whether DP1 contributes to E2F1 induction of apoptosis in WI-38 cells. Ectopic expression of DP1, which enhanced endogenous *CDC6* gene expression by ectopic E2F1 (Fig. 3A), did not significantly increase E2F1 induction of apoptosis at low or high MOI of Ad-E2F1 (Fig. 6G and H). Taken together, these results indicate that, while cell cycle progression induced by both physiological and deregulated E2F is dependent on DP1, apoptosis induced by deregulated E2F is independent of DP1 in human normal fibroblasts.

## Discussion

It is generally accepted that the E2F heterodimeric partner DP contributes to E2F regulation of genes involved not only in cell cycle progression but also in apoptosis^2,24^. In this study, however, we showed that, in contrast to *CDC6* gene expression and cell cycle progression, deregulated E2F induction of *ARF* gene expression and apoptosis is independent of DP family members in human normal fibroblasts. ShRNA-mediated knockdown of DP1 and DP2 expression did not inhibit deregulated E2F-induced *ARF* gene expression or apoptosis, while DP1 knockdown alone compromised E2F-induced *CDC6* gene expression and cell cycle progression. DP1 knockdown also did not affect interaction of ectopic E2F1 with the ARF promoter or E2F1-mediated ARF promoter activation, while it reduced binding of ectopic E2F1 to and E2F1-mediated activation of the CDC6 promoter. Furthermore, over-expression of DP1 did not significantly affect E2F1 binding to or activation of ARF promoter, whereas it enhanced E2F1 in binding to and activation of the CDC6 promoter. Taken together, these results suggest that deregulated E2F-induced *ARF* gene expression and apoptosis is independent of DP family members in human normal fibroblasts. This is in sharp contrast to E2F-mediated promotion of cell cycle progression through the regulation of cell cycle-related gene expression that is dependent on DP.

The mechanism, by which E2F regulates genes involved in opposite cell fates of cell proliferation and apoptosis, is not fully understood. The threshold model has been proposed to explain the differential regulation of these processes by E2F, based on the observation that apoptosis caused by *RB1* knockout is alleviated by combinatorial knockout of *E2F1* or *E2F3*^2^. In this model, E2F regulation of genes involved in cell proliferation and apoptosis is a function of total activity of activator E2Fs freed from RB. When such activity exceeds an initial threshold, E2F induces genes involved in cell proliferation, whereas E2F activation of genes involved in apoptosis requires a second, higher threshold. Accordingly, when total activity of activator E2Fs is reduced by DP knockdown, induction of pro-apoptotic gene expression and apoptosis should be compromised before that of proliferation-related genes and cell cycle progression. However, our results are opposite to this expectation, as DP knockdown suppressed induction of *CDC6* gene expression and cell cycle progression but not induction of *ARF* gene expression or apoptosis. Moreover, DP1 knockdown reduced binding of ectopically expressed E2F1 to the CDC6 promoter but not to the ARF promoter. These results indicate that E2F regulation of the *ARF* and *CDC6* genes is not solely determined by differential thresholds of total activity of activator E2Fs.

The E2F responsive sequence of ARF promoter (GC repeat) is distinct from that of proliferation-related genes (TTT^C^/_G_^G^/_C_CGC). Similarly, the E2F responsive element of the BIM promoter, which is also specifically activated by deregulated E2F activity, is also composed of GC repeats^25^. Moreover, in ChIP assays, binding of E2F1 to the GC repeat was only detected upon ectopic expression of E2F1 or E1a and not detected under normal growth conditions^17,25^. Thus, these pro-apoptotic tumor suppressor genes are only activated when deregulated E2F1 binds to the distinct response elements. In addition, while binding of physiologically induced E2F to a canonical response element requires DP1, binding of deregulated E2F1 to this atypical sequence is independent of DP proteins. Taken together, these observations imply that deregulated E2F is functionally distinct from its physiologically activated counterpart.

We considered three possibilities to explain the lack of effect of DP expression on E2F activation of the *ARF* gene. Firstly, a low level of E2F1 is sufficient to activate the *ARF* gene and consequently minimal DP expression is sufficient. However, in ChIP assays, similar intensities of bands was observed for *ARF* and *CDC6* genes at the same PCR amplification cycles, suggesting that the similar amounts of E2F1 are bound to the *ARF* and *CDC6* genes. Secondly, E2F utilizes a distinct partner, other than DP, to bind to the ARF promoter, or thirdly, deregulated E2F1 binds to ARF promoter by itself. Electrophoretic mobility shift assays demonstrated that purified GST-E2F1 alone could bind to EREA (Fig. 3D). Moreover, binding of GST-E2F1 to EREA was not significantly affected by the addition of GST-DP1, while the interaction of GST-E2F1 with a typical E2F target site was clearly enhanced in the presence of GST-DP1. These results suggest that E2F1 alone is sufficient for binding to EREA, and that, at least *in vitro*, this interaction is not dependent on DP1. Accordingly, neither knockdown nor ectopic expression of DP1 significantly affected binding of ectopically expressed E2F1 to the ARF promoter *in vivo* as shown by the ChIP assay (Figs 2C and 3E). These results support the second and third possibilities that deregulated E2F1 recognizes EREA independently of DP proteins in human normal fibroblasts. In this respect, it is interesting to note the results of genome-wide analysis of over-expressed E2F1 binding sites *in vivo*^26^. ChIP assay with an E2F1 mutant lacking the marked box domain, which is critical for interaction of E2F1 with DP1, resulted in similar binding sites compared to that of wild type E2F1, suggesting that DP1 is not an obligate interaction partner for over-expressed E2F1 binding to DNA *in vivo*. Moreover, the ChIP assays, using anti-DP1 or HA-DP epitope tag antibodies, were unable to demonstrate binding of DP1 to any E2F target^26^, suggesting that DP1 is either masked in the E2F1-DP1 complex or DP1 is not bound with E2F1 at response elements.

In this study, we analyzed the *CDC6* and *ARF* genes as representatives of growth-related and pro-apoptotic genes, respectively. It is well established that growth-related E2F targets contain canonical E2F binding sites (TTT^C^/_G_^G^/_C_CGC) usually within 100 bp from the transcriptional start sites^1–3^. In contrast, ARF gene contains atypical E2F binding sequence (GC repeat)^17^. It is interesting to note that, in the genome-wide analysis of over-expressed E2F1 binding sites *in vivo*^26^, only 12% of the identified sites are typical E2F binding sites (TTT^C^/_G_^G^/_C_CGC), with the remainder contain CGCGC motifs similar to the atypical E2F binding sequence of the *ARF* and *BIM* genes. The genes identified in that study contain not only growth-related and pro-apoptotic genes but also genes involved in DNA damage response, DNA repair, ubiquitin-like conjugation, mRNA processing, mRNA splicing and other cellular processes. We have also identified genes, which are regulated by E2F in the same manner as the *ARF* gene, such as *p27*^*Kip1*^, *TAp73*, *BIM*, *RASSF1*, *PPP1R13B*, *JMY*, *MOAP1*, *RBM38*, *ABTB1*, *RBBP4* and *RBBP7*^25,27,28^. Among these, *p27*^*Kip1*^, *TAp73* and *ABTB1* contain typical E2F binding sites, but located more than 100 bp upstream from the transcriptional start sites, and *BIM*, *JMY*, *MOAP1*, *RBBP4* and *RBBP7* contain CGCGC. Although all of the genes identified by over-expression of E2F1 may not be *bona fide* targets of endogenous deregulated E2F, we speculate that many of the genes containing CGCGC motifs may be regulated in the same manner as the *ARF* gene.

In summary, our results show that deregulated E2F induction of *ARF* gene expression and apoptosis is independent of DP family members in contrast to *CDC6* gene expression and cell cycle progression. Moreover, DP knockdown suppressed induction of *CDC6* gene expression and cell cycle progression but not induction of *ARF* gene expression or apoptosis, which contradicts expectation from the threshold model. The differential regulation of growth-related genes and pro-apoptotic genes by E2F is not sufficiently explained by the total amount of free E2Fs, suggesting an additional layer of regulatory mechanism such as qualitative difference of E2F, which awaits to be elucidated.

## Methods

### Cell culture and cell proliferation analysis

Normal human fibroblast WI-38 cells (RIKEN Bioresource Center Cell Bank) and 293A cells (Invitrogen) were cultured in Dulbecco’s modified Eagle medium containing 10% FCS. Cells were trypsinized and counted at indicated time points after serum stimulation. Cell counting was performed three times and values are shown as means ± SD.

### Plasmid and recombinant adenovirus constructions

pARF-Luc(-736), pCDC6-Luc, pCMV-β-gal, the DP2 expression vector pCMV-DP2, the GST-E2F1 expression vector pGEX-2TK-RBAP-1, the GST-DP1 expression vector pGST-DP1, the Flag-tagged DP3 expression vector pCMV3-FLAG-DP3, the E2F1 expressing adenovirus Ad-E2F1, and the E1a expressing adenovirus Ad-12SE1A have been previously described^17,18,29–31^. *E2F1* cDNA from pDCE2F and *DP1* cDNA from pCMV-DP1^23^ were cloned into pENTR-CMV, which was generated by subcloning a CMV promoter-driven expression cassette from pcDNA3 (Invitrogen) into pENTR-D-TOPO (Invitrogen), creating expression entry vectors pENTR-E2F1 and pENTR-DP1, respectively. The shRNA expression entry vectors were constructed by inserting double-stranded oligonucleotides including the target sequence into pENTR-U6, which was generated by subcloning the human U6 promoter from pSilencer 2.0-U6 (Ambion) into pENTR-D-TOPO. Nineteen-bp DNA sequences for RNA interference against human *DP1* mRNA (nucleotides 396–414 (for shDP1-2) and 679–697 (for shDP1-3) of human *DP1* cDNA; GenBank accession number: NM_007111), against human *DP2* mRNA (nucleotides 988–1006 of human DP2 cDNA; GenBank accession number: U18422) and against human *ARF* mRNA (nucleotides 114–132 of human *ARF* cDNA; GenBank accession number: NM_058195) were selected based on a published report^32^. Recombinant adenoviruses were produced using the entry vectors and ViraPower Adenoviral Expression System (Invitrogen) according to the supplier’s protocol.

### Infection with recombinant adenoviruses and FACS analysis

Infection with recombinant adenoviruses proceeded as previously described^17^. For FACS analysis, cells were harvested with any floating cells. To measure the percentage of cells with a sub-G_1_ DNA content, harvested cells were fixed with lysing solution (BD Biosciences), stained with propidium iodide, treated with RNase, and analyzed by FACSCalibur (BD Biosciences). To detect a cell surface change in the apoptotic process, annexin V staining was performed using TACS^TM^ Annexin V-FITC (R & D Systems) according to the manufacturer’s protocol and the percentage of cells stained with annexin V was analyzed by FACSCalibur. To measure the percentage of cells incorporating BrdU, 10 μM BrdU was added to the cell culture at 20 h before harvest. Harvested cells were then fixed, permeabilized, and incubated with a BrdU antibody (1170379; Roche). The cells were then incubated with FITC labeled antibody to mouse immunoglobulin (Dako Cytomation; F0232) and analyzed by FACSCalibur. All assays were performed at least three times and values are shown as means ± SE. Statistical significance of the difference was determined with student’s *t*-test.

### Transfection and reporter assay

Luciferase assays proceeded as previously described^17^. Reporter activities were normalized to β-galactosidase activities as an internal control. All assays were repeated at least three times in duplicate and values are shown as means ± SD. Statistical significance of the difference between the mean was determined with student’s *t*-test.

### ChIP assay

ChIP assay was performed as previously described^17^. Specific primer sets were: ARF promoter, 5′-ggcggtaggcgggagggagaggaa-3′ and 5′-cgtgagccgcgggatgtgaacca-3′, human β-actin promoter, 5′-ctccctcctcctcttcctcaatct-3′ and 5′-cgtcgcgccgctgggttttat-3′, and CDC6 promoter as described^33^. Antibodies for immunoprecipitating protein-DNA complexes were anti-E2F1 (KH95), anti-E2F4 (C-108), and anti-p50 (NLS) (all from Santa Cruz).

### Immunoblotting

Immunoblotting was carried out as previously described^17^. Antibodies used were anti-DP1 (TFD10; BD Biosciences Pharmingen), anti-DP2 (G-12; Santa Cruz), anti-E1a (M58; BD Biosciences Pharmingen), anti-E2F1 (KH95; Santa Cruz), anti-FLAG (M2; Sigma), anti-p14^ARF^ (Ab-1; NeoMarkers), anti-p53 (DO-1; Santa Cruz), and anti-α-tubulin (DM1A; Oncogene Research Products).

### Isolation of mRNA and RT -PCR

Isolation of mRNA and RT-PCR were performed as previously described^17^. Specific primer sets for PCR were: *DP1*, 5′-ttagtcccgggaaaggcgtggtgt-3′ and 5′-cgccgtcttatgtttttctggtca-3′, *DP2*, 5′-ataaccatttggctgctgatt-3′ and 5′-ctgggcccgcttctgctttat-3′, *DP3*, 5′-ctcactgaagctaacgaagaactc-3′ and 5′-tcatggaaagacggcacag-3′, *Bax*, 5′-ggttgtcgcccttttctact-3′ and 5′-tgagcactcccgccacaa-3′, those for *CDC6*, *ARF*, and *GAPDH* were as described^17,34^. The PCR products were resolved by electrophoresis on 2% agarose gels and visualized by ethidium bromide staining.

### Gel mobility shift assay

Gel mobility shift assay was carried out as previously described^17^. A DHFR promoter fragment containing typical E2F sites and EREA were used as probes. The adenovirus E2 enhancer and its mutants were used as competitors.

## Electronic supplementary material

Supplementary Figures
