# Supplementary material for: Differential requirement for dimerization partner DP between E2F-dependent activation of tumor suppressor and growth-related genes
Source: Sci Rep. 2018 May 31;8:8438. doi: 10.1038/s41598-018-26860-0 (PMC5981219; doi:10.1038/s41598-018-26860-0)
Supplement: Supplementary file 1 — Supplementary Figures [file 41598_2018_26860_MOESM1_ESM.pdf]

Differential requirement for dimerization partner DP between E2F-dependent activation of tumor suppressor and growth-related genes

Hideyuki Komori[1], Yasuko Goto[2], Kenta Kurayoshi[2], Eiko Ozono[3], Ritsuko Iwanaga[4], Andrew P. Bradford[5], Keigo Araki[2] and Kiyoshi Ohtani[2]

[1]Life Sciences Institute, University of Michigan,
210 Washtenaw Avenue, Ann Arbor, MI 48109-2216, USA

[2]Department of Biomedical Chemistry, School of Science and Technology,
Kwansei Gakuin University,
2-1 Gakuen, Sanda, Hyogo, 669-1337, Japan

[3]Chromosome Replication Lab, The Francis Crick Institute,
Midland Road, NW1 1AT, UK

[4]Department of Craniofacial Biology,
University of Colorado School of Dental Medicine, Anschutz Medical Campus,
12801 East 17th Avenue, Aurora, CO 80045, USA

[5]Department of Obstetrics and Gynecology,
University of Colorado School of Medicine, Anschutz Medical Campus,
12800 East 19th Avenue, Aurora, CO 80045, USA

Correspondence:
Kiyoshi Ohtani
Department of Biomedical Chemistry, School of Science and Technology,
Kwansei Gakuin University,
2-1 Gakuen, Sanda, Hyogo, 669-1337, Japan
Phone: +81-79-565-7219
Fax: +81-3-79-565-7219
E-mail: btm88939@kwansei.ac.jp

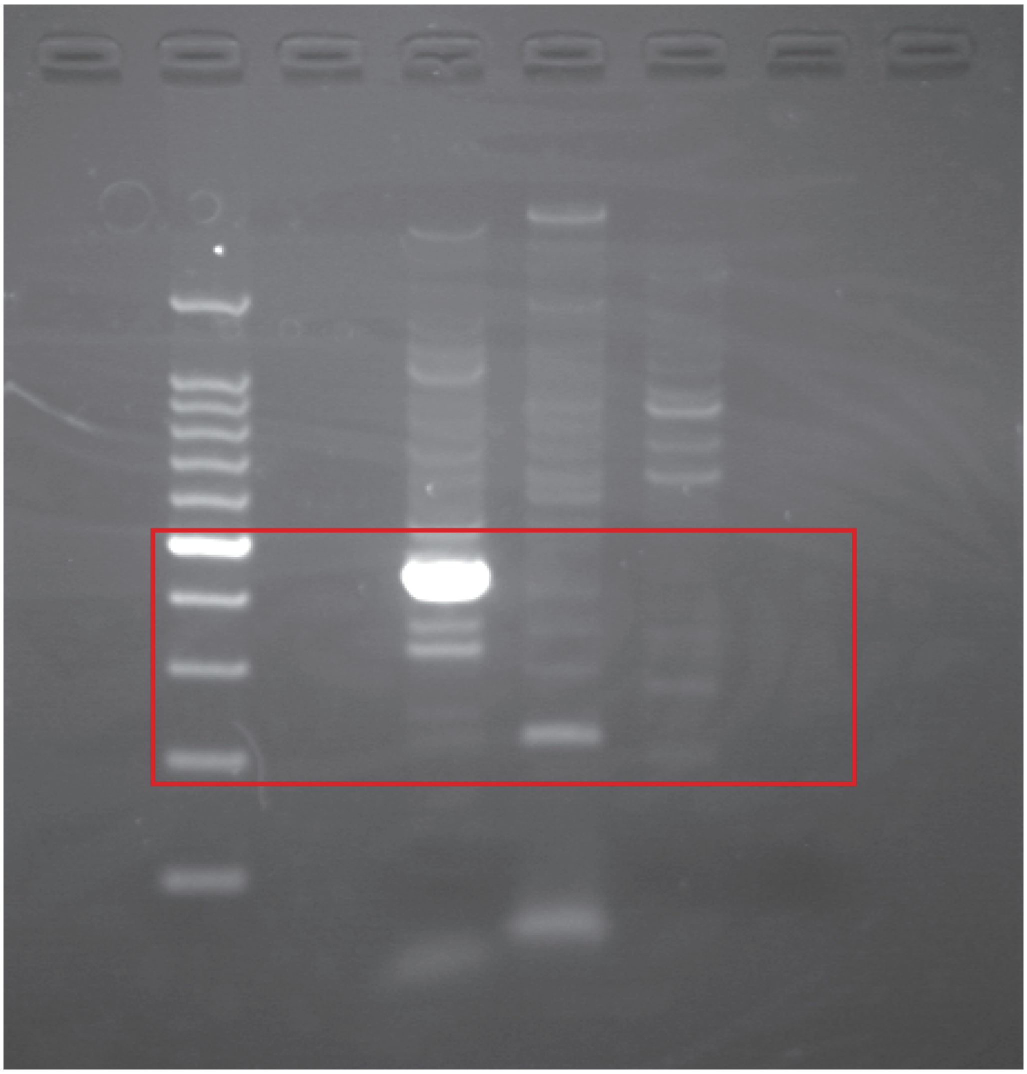

Supplementary Figure S1

Full-length gel of PCR products illustrated in Figure 1(A).

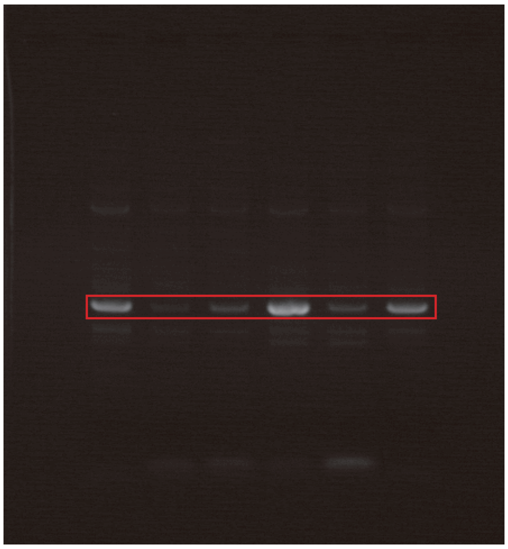

DP1

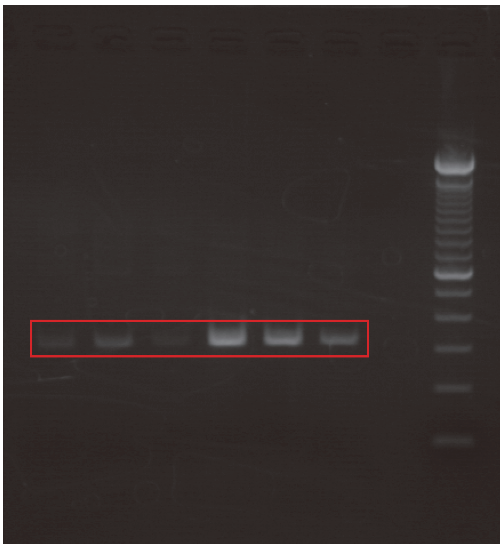

CDC6

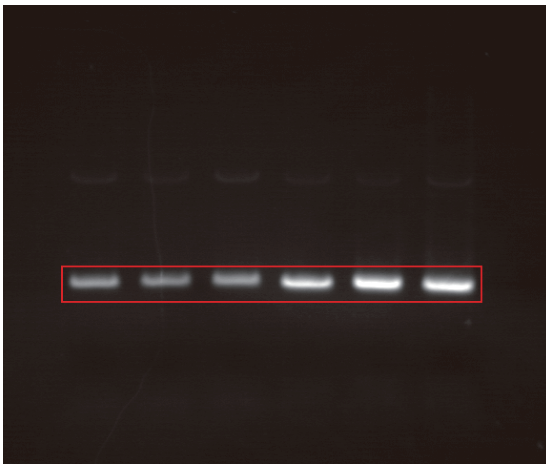

ARF

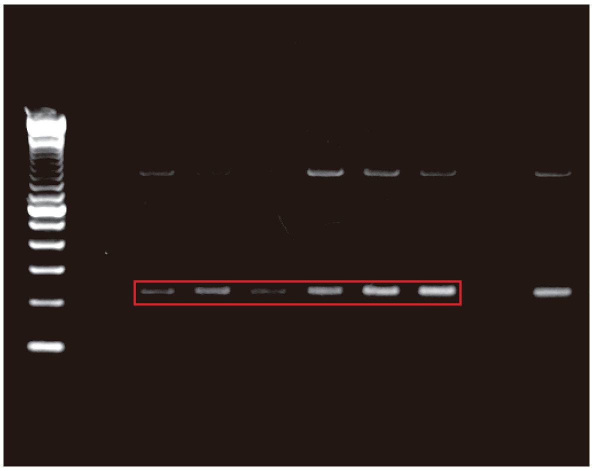

Bax

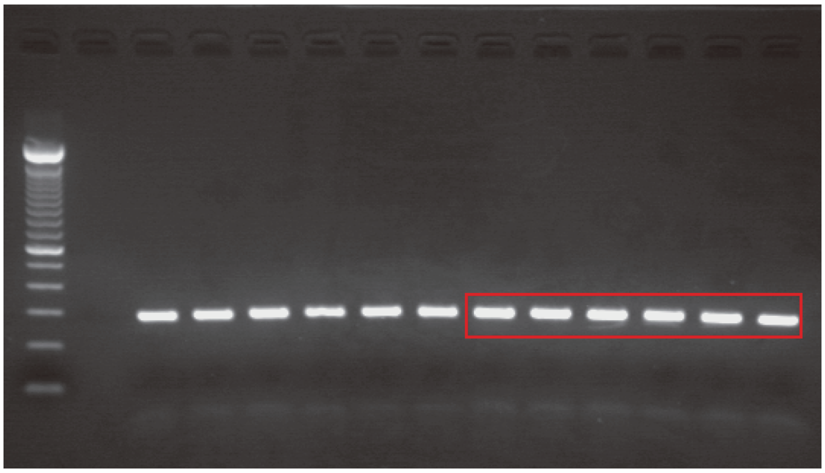

GAPDH

Supplementary Figure S2

Full-length gels of indicated PCR products illustrated in Figure 1(C).

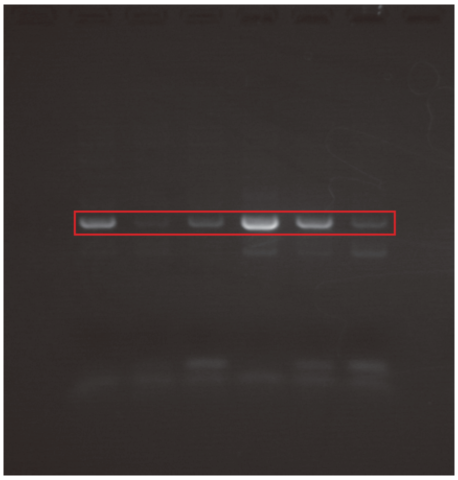

DP1

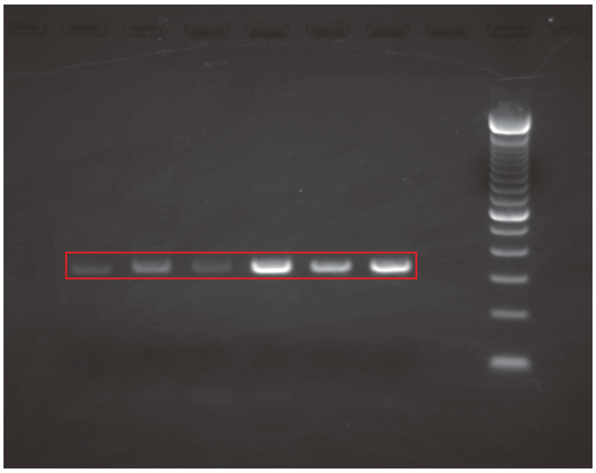

CDC6

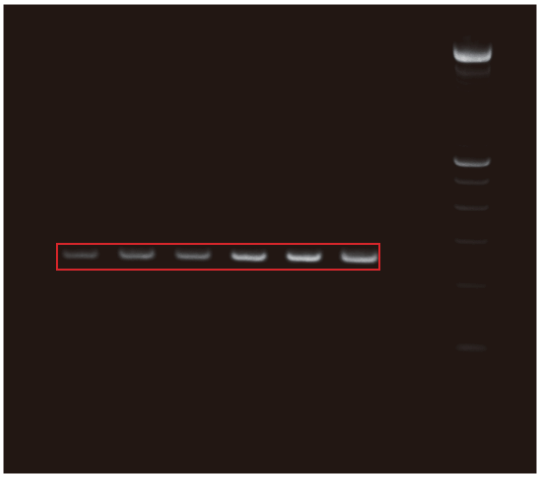

ARF

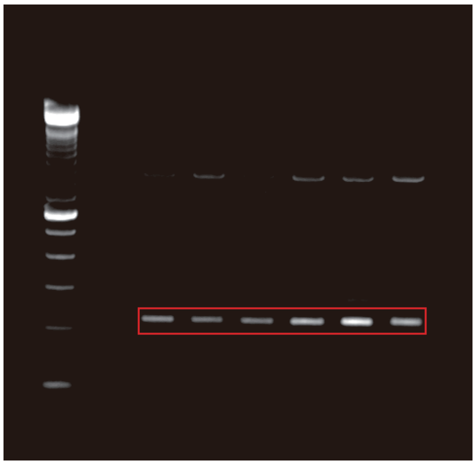

Bax

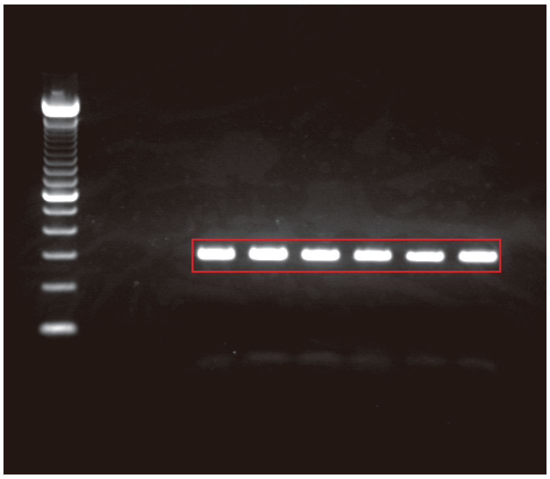

GAPDH

Supplementary Figure S3

Full-length gels of indicated PCR products illustrated in Figure 1(D).

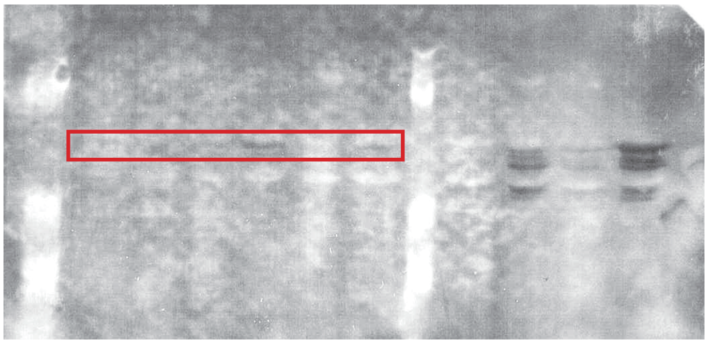

DP1

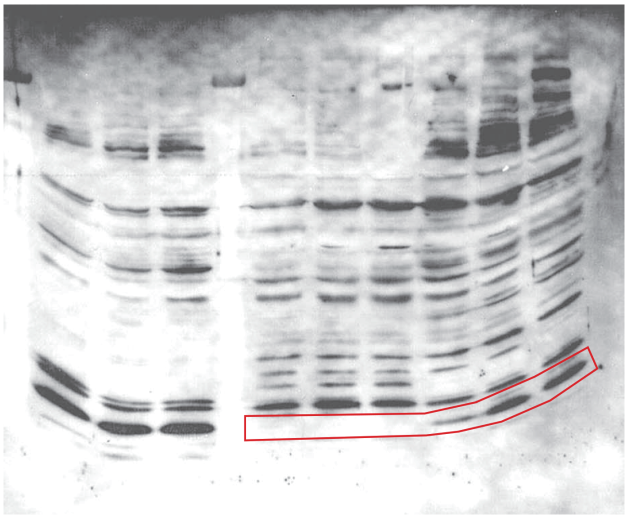

ARF

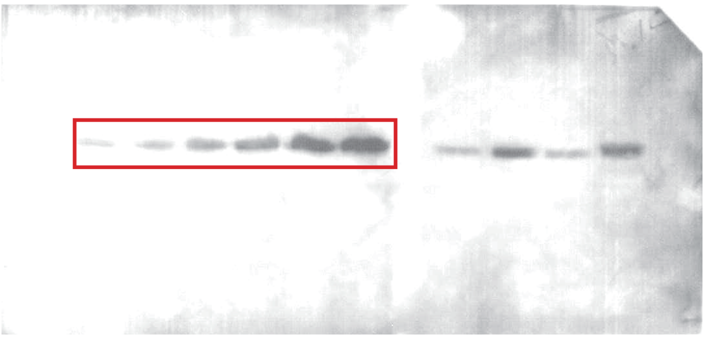

p53

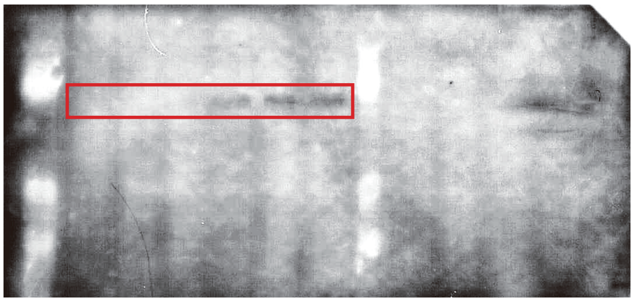

E2F1

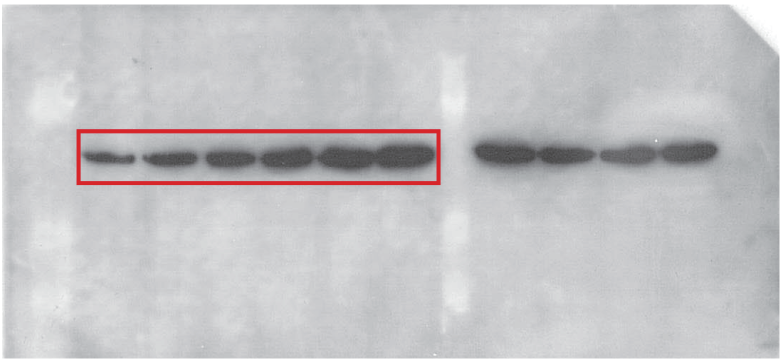

α-tubulin

Supplementary Figure S4

Full-length blots of indicated proteins illustrated in Figure 1(E).

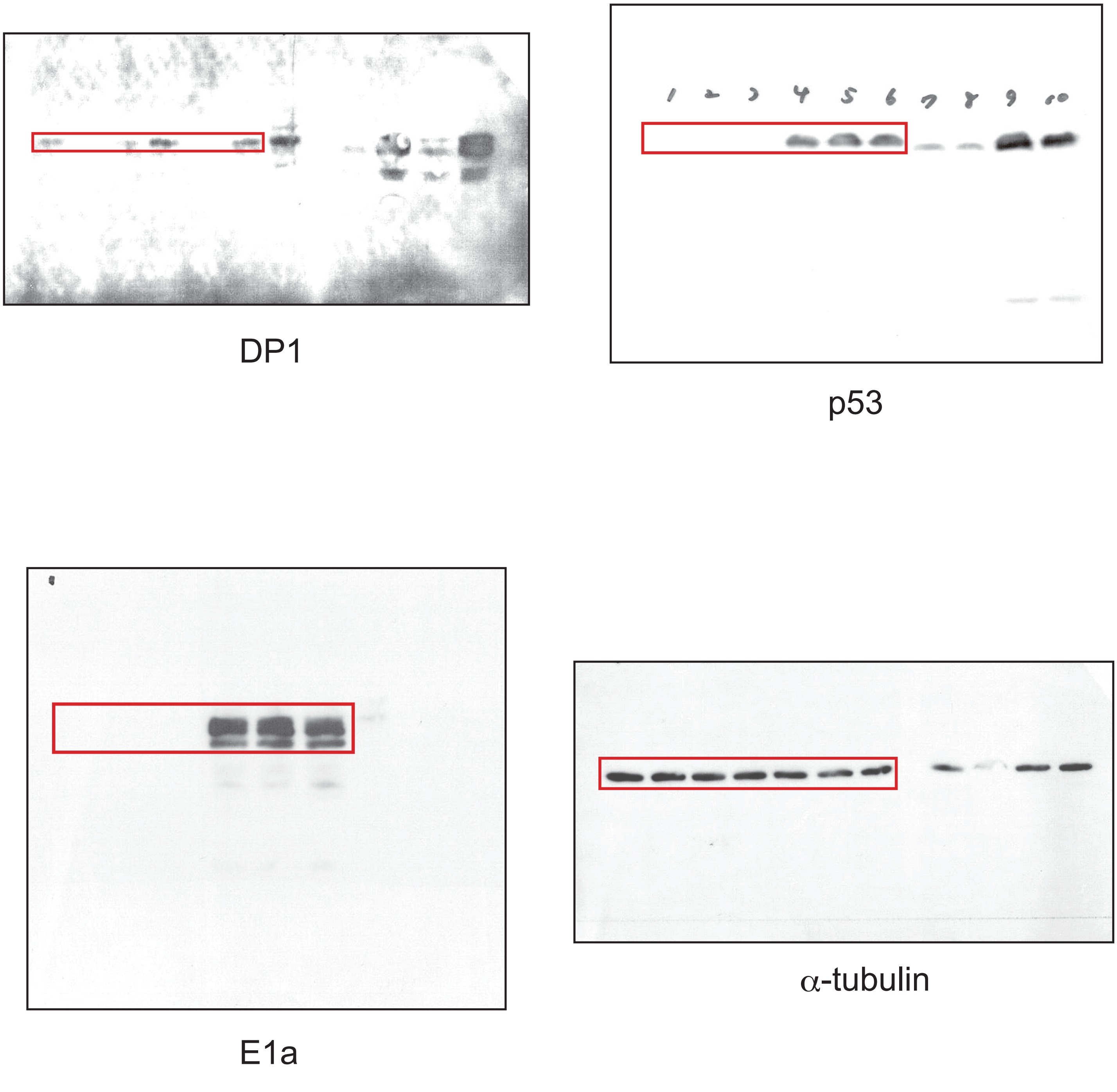

Supplementary Figure S5

Full-length blots of indicated proteins illustrated in Figure 1(F).

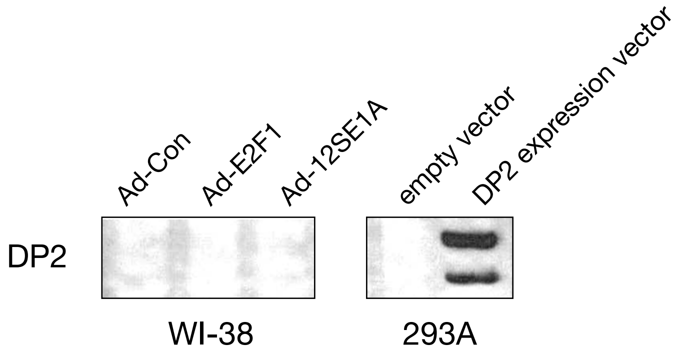

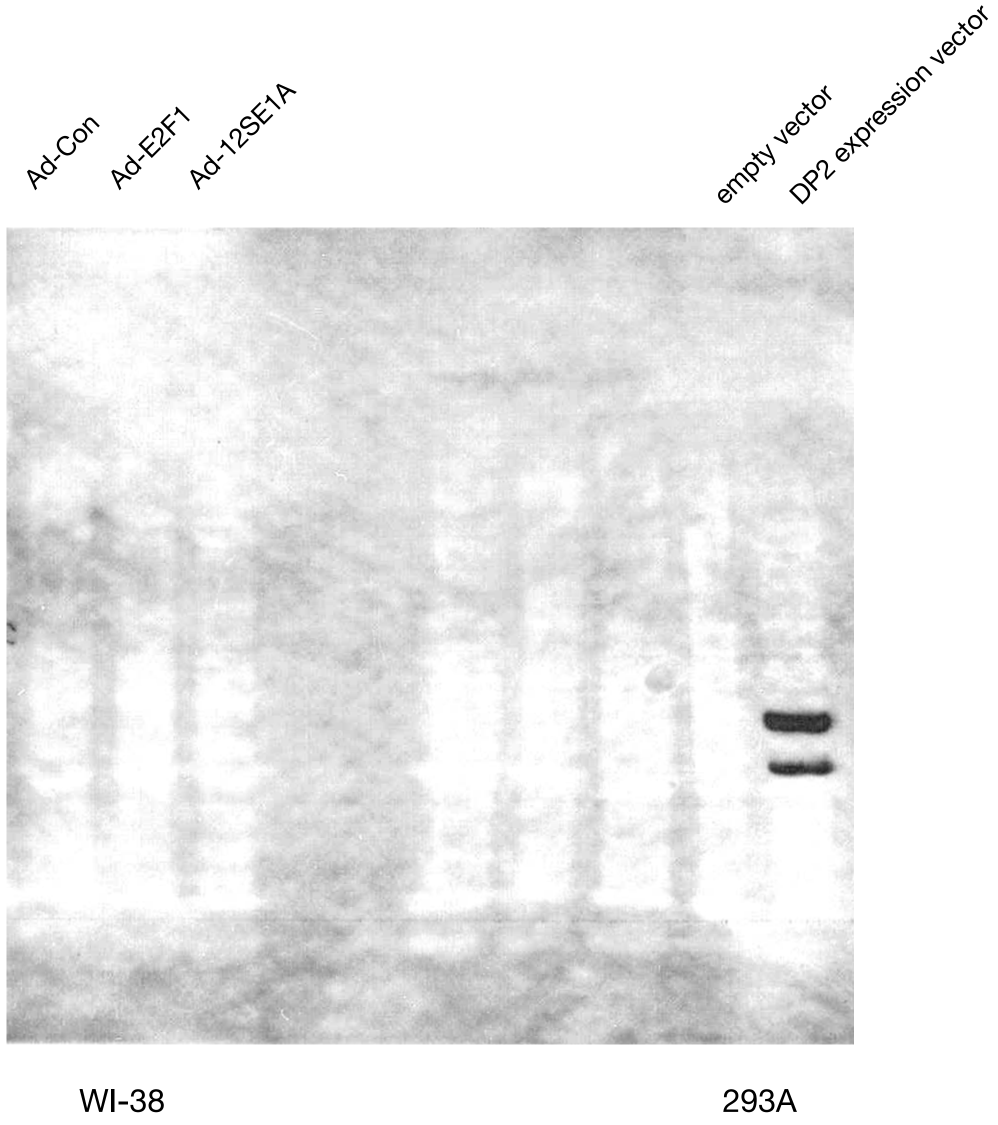

Supplementary Figure S6

Expression of DP2 protein was not detected by immunoblot analysis even with ectopic expression of E2F1 or E1a. Lower panel is full-length blot of upper panel.

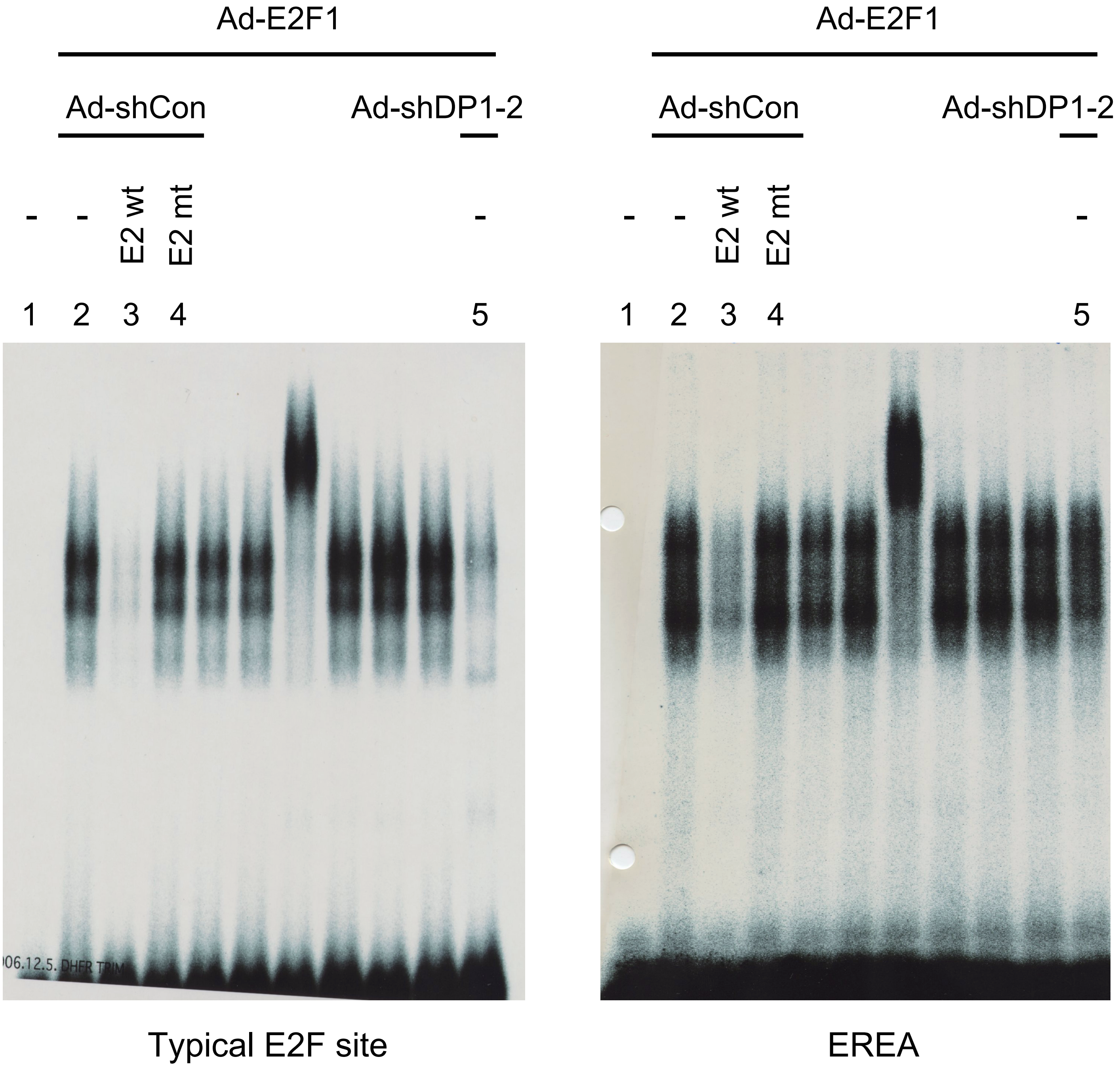

Supplementry Figure S7
Full-length gels of gel mobility shift assays illustrated in Figure 2 (B).

ARF

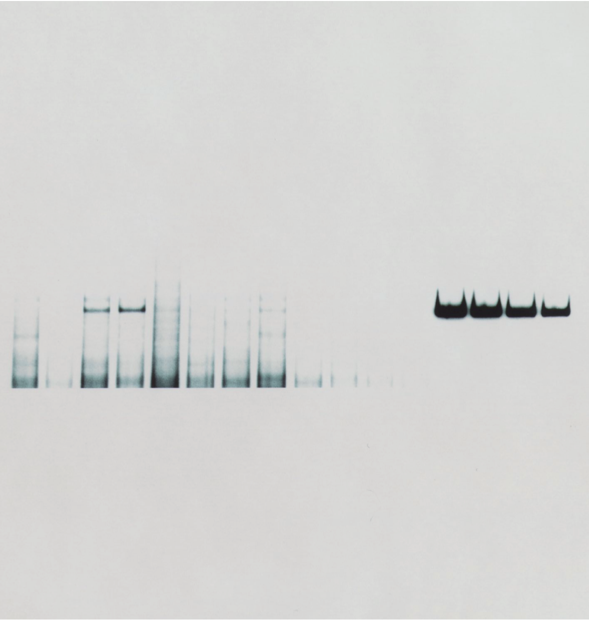

CDC6

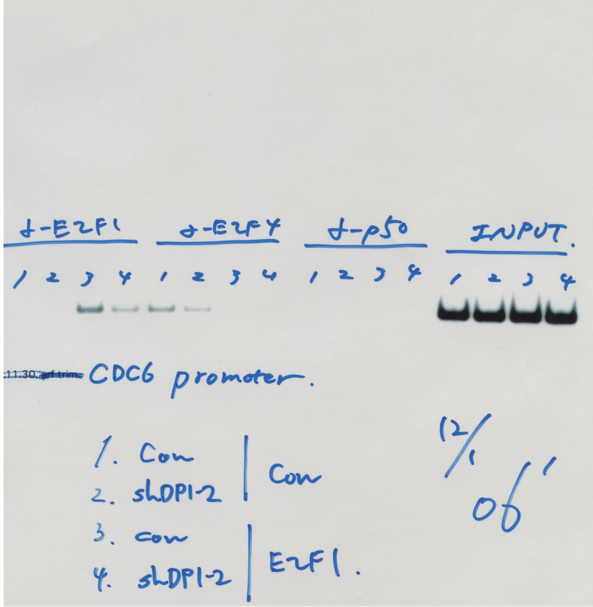

β-*actin*

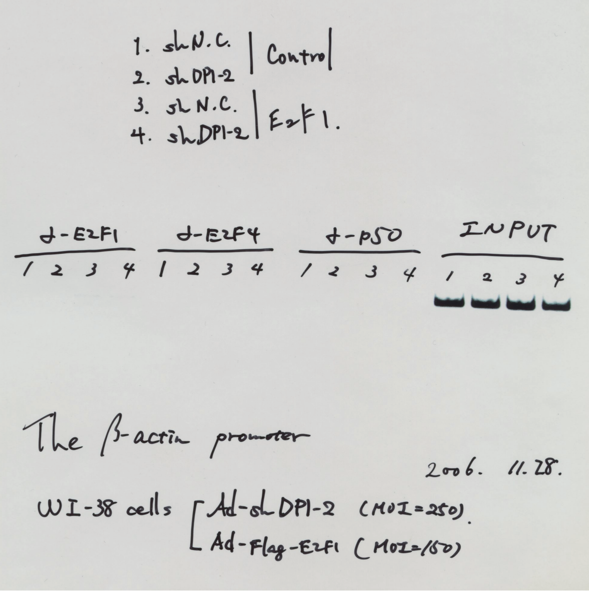

Supplementary Figure S8

Full-length gels of ChIP analysis illustrated in Figure 2(C).

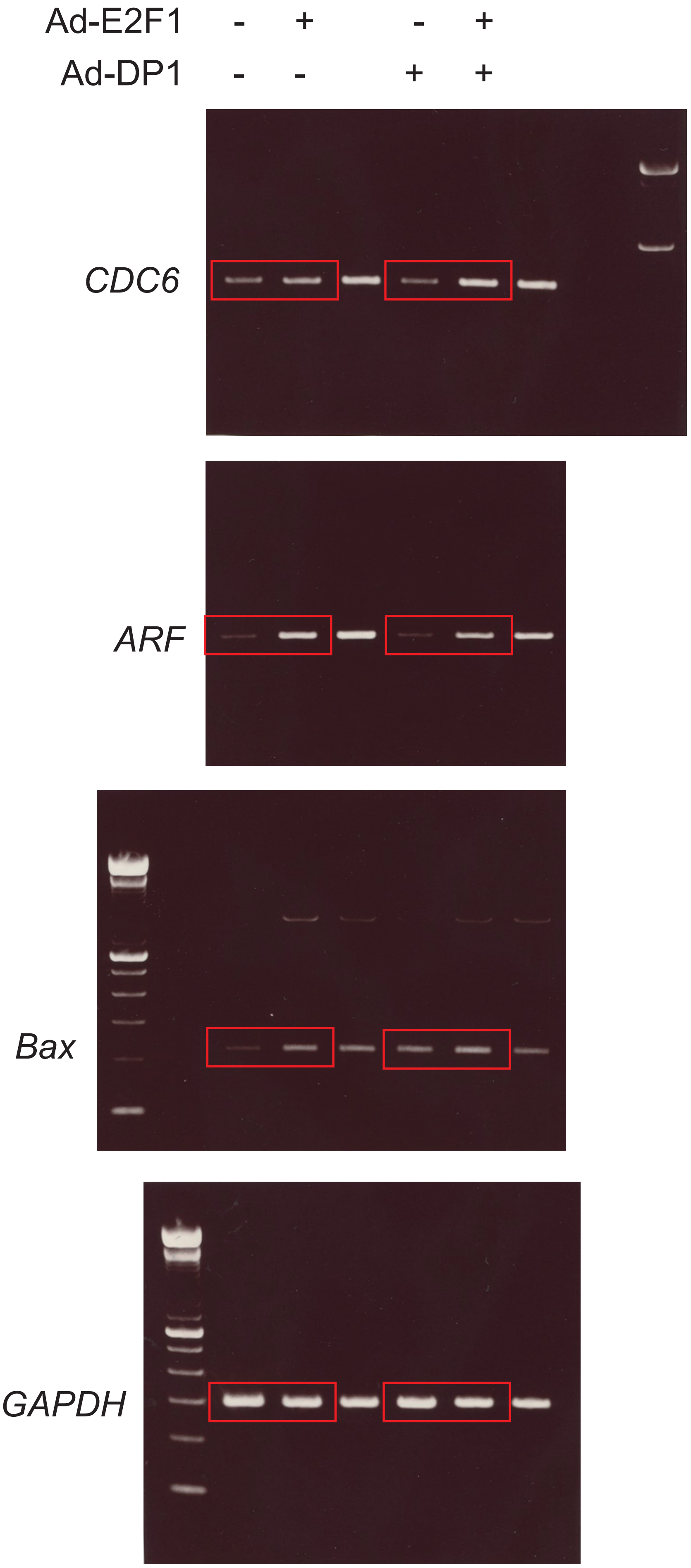

Supplementary Figure S9

Full-length gels of indicated PCR products illustrated in Figure 3(A).

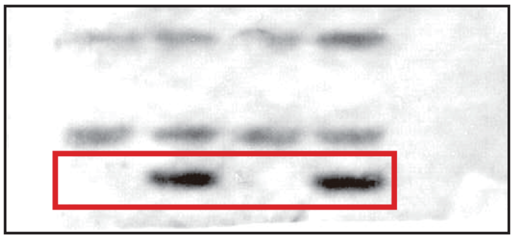

ARF

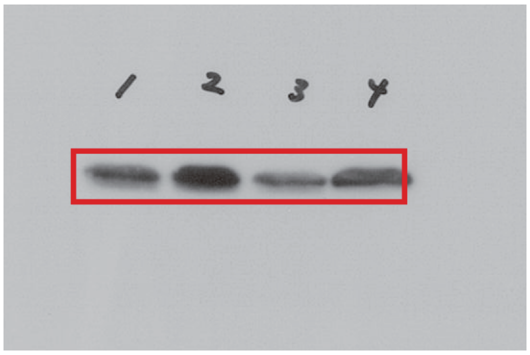

p53

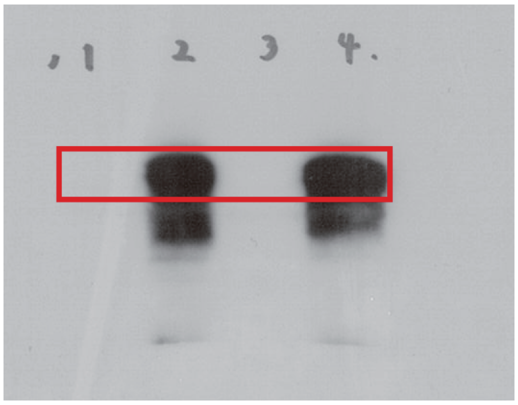

E2F1

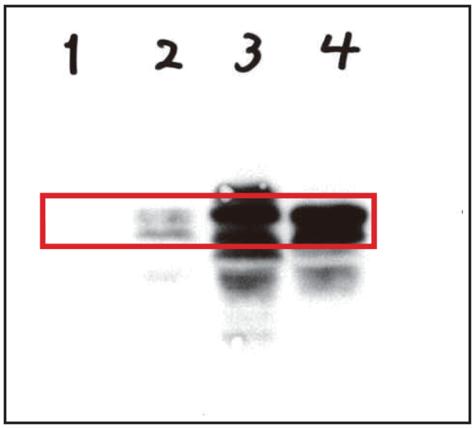

DP1

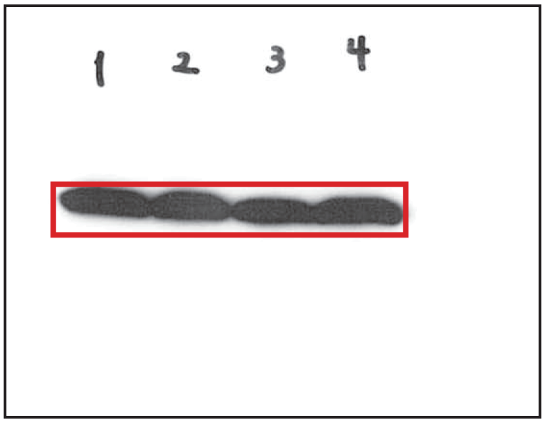

α-tubulin

Supplementary Figure S10

Full-length blots of indicted proteins illustrated in Figure 3(B).

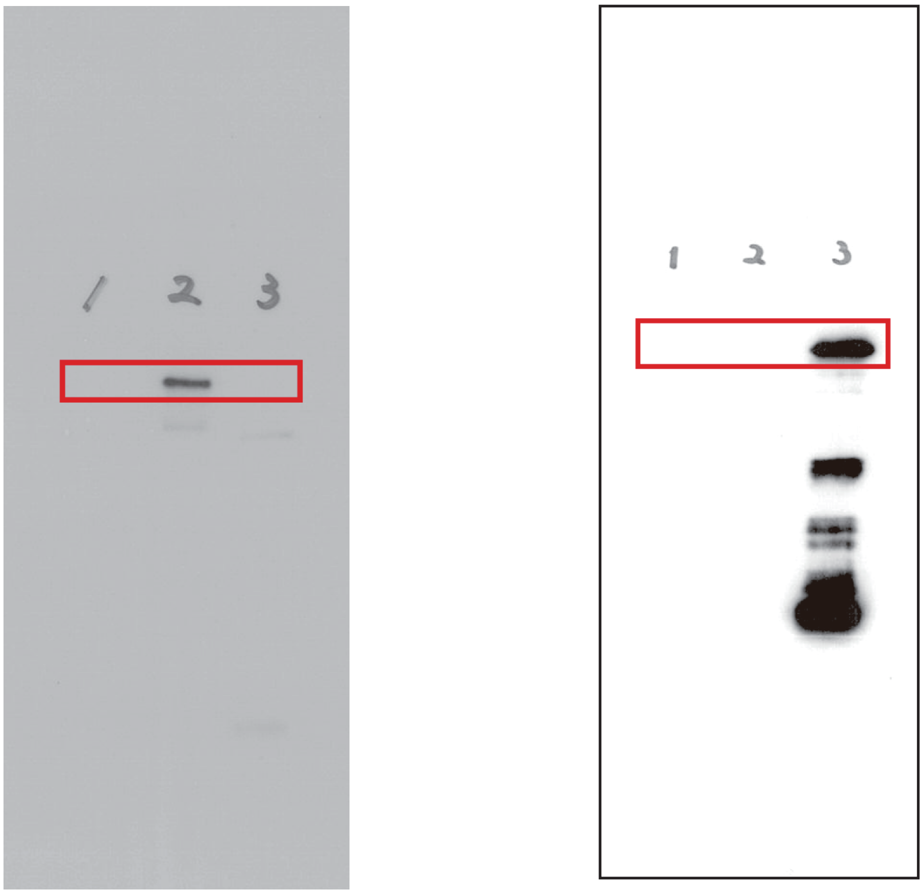

GST-E2F1 GST-DP1

Supplementary Figure S11

Full-length blots of purified GST-E2F1 and GST-DP1 illustrated in Figure 3(D).

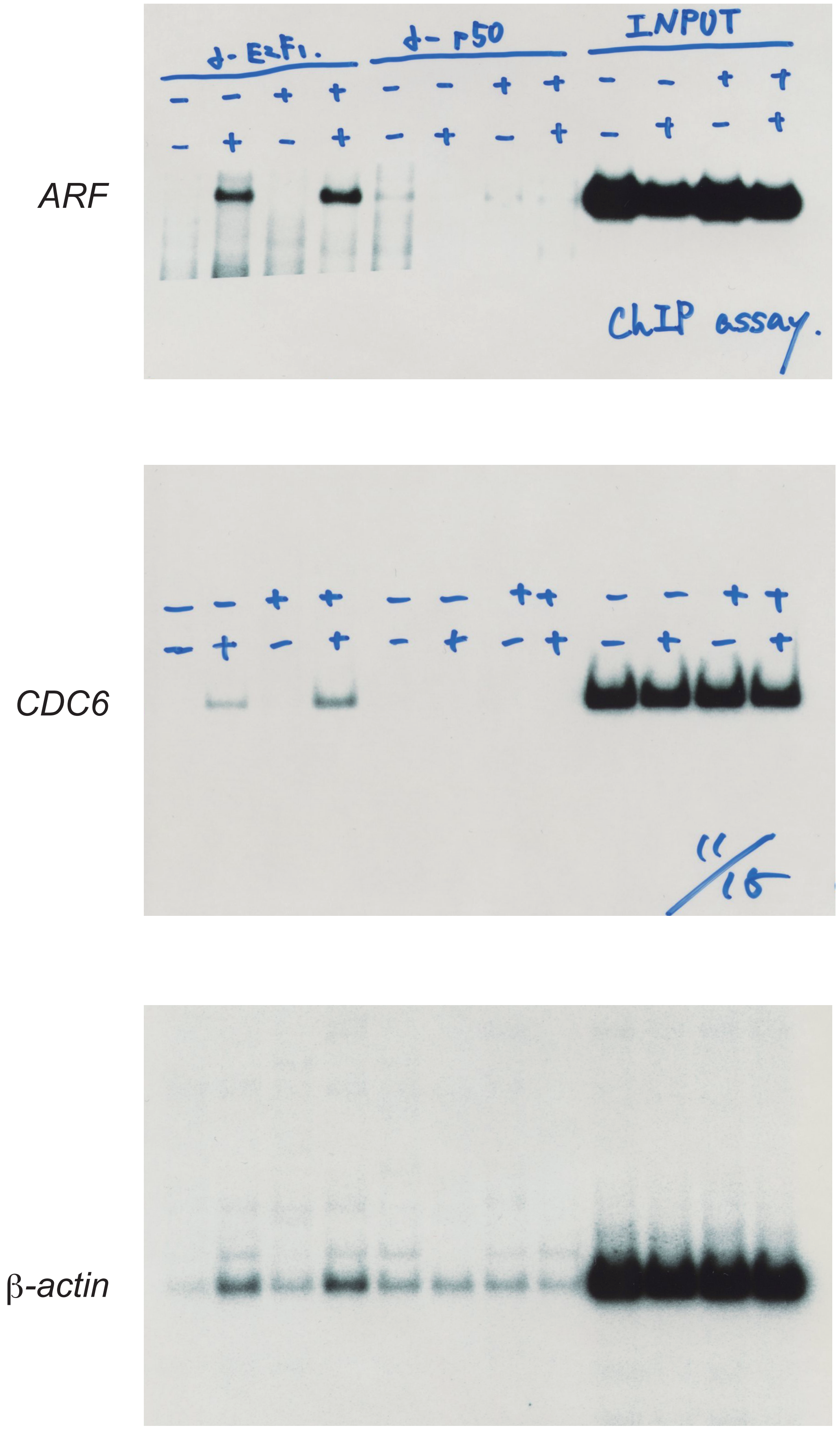

Supplementary Figure S12

Full-length gels of ChIP analysis illustrated in Figure 3(E).

DP1

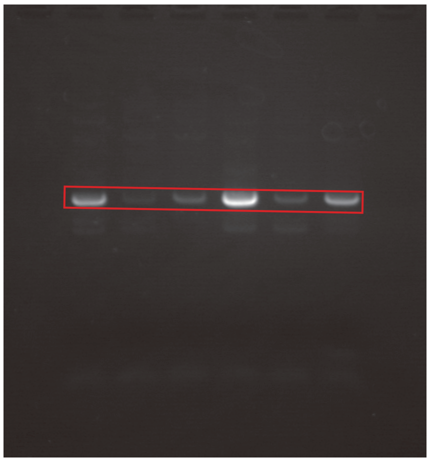

DP2

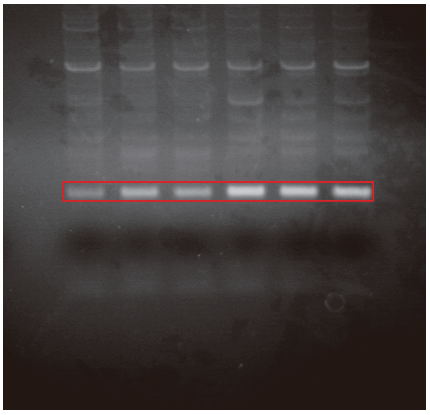

DP3

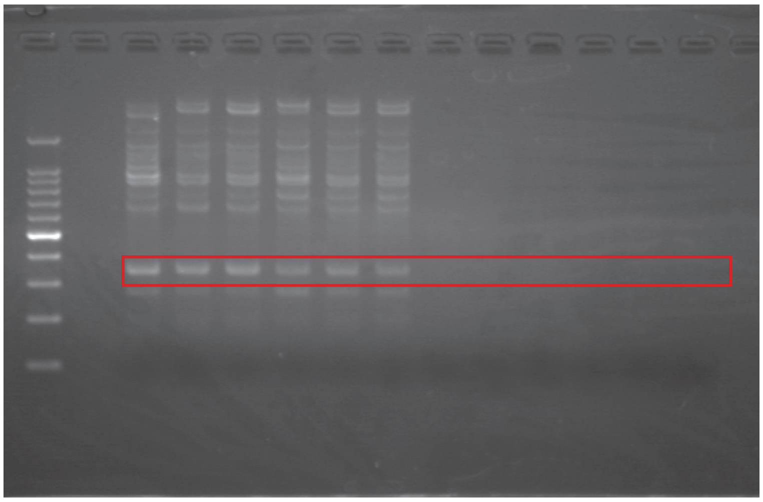

GAPDH

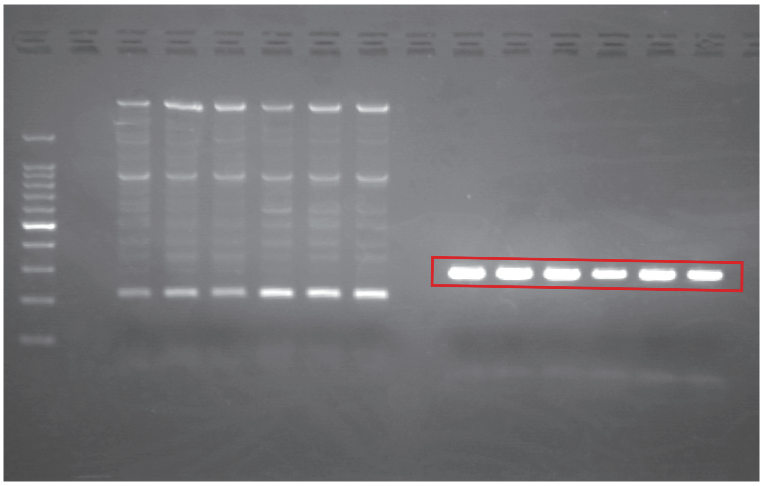

Supplementary Figure S13

Full-length gels of indicated PCR products illustrated in Figure 4(A).

DP1

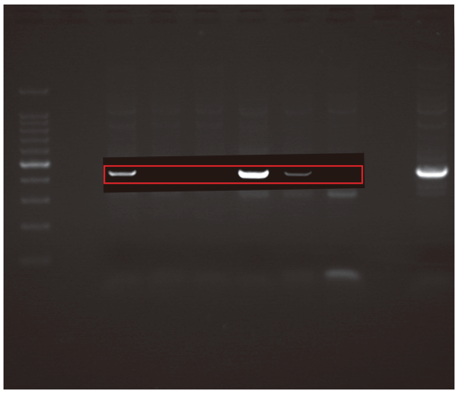

DP2

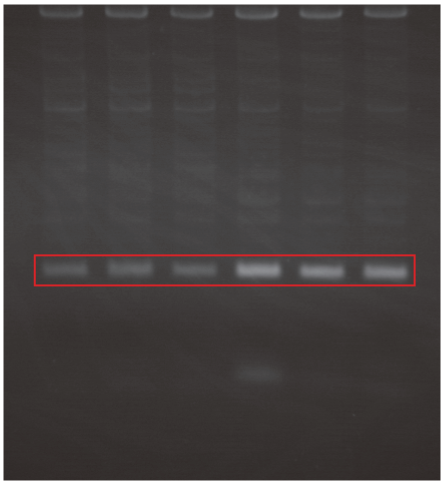

DP3

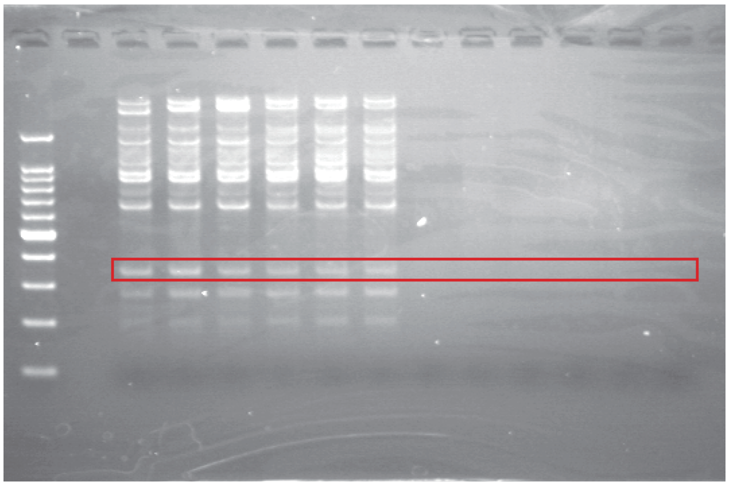

GAPDH

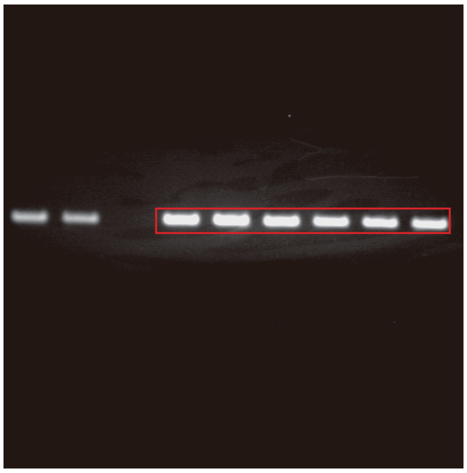

Supplementary Figure S14

Full-length gels of indicated PCR products illustrated in Figure 4(B).

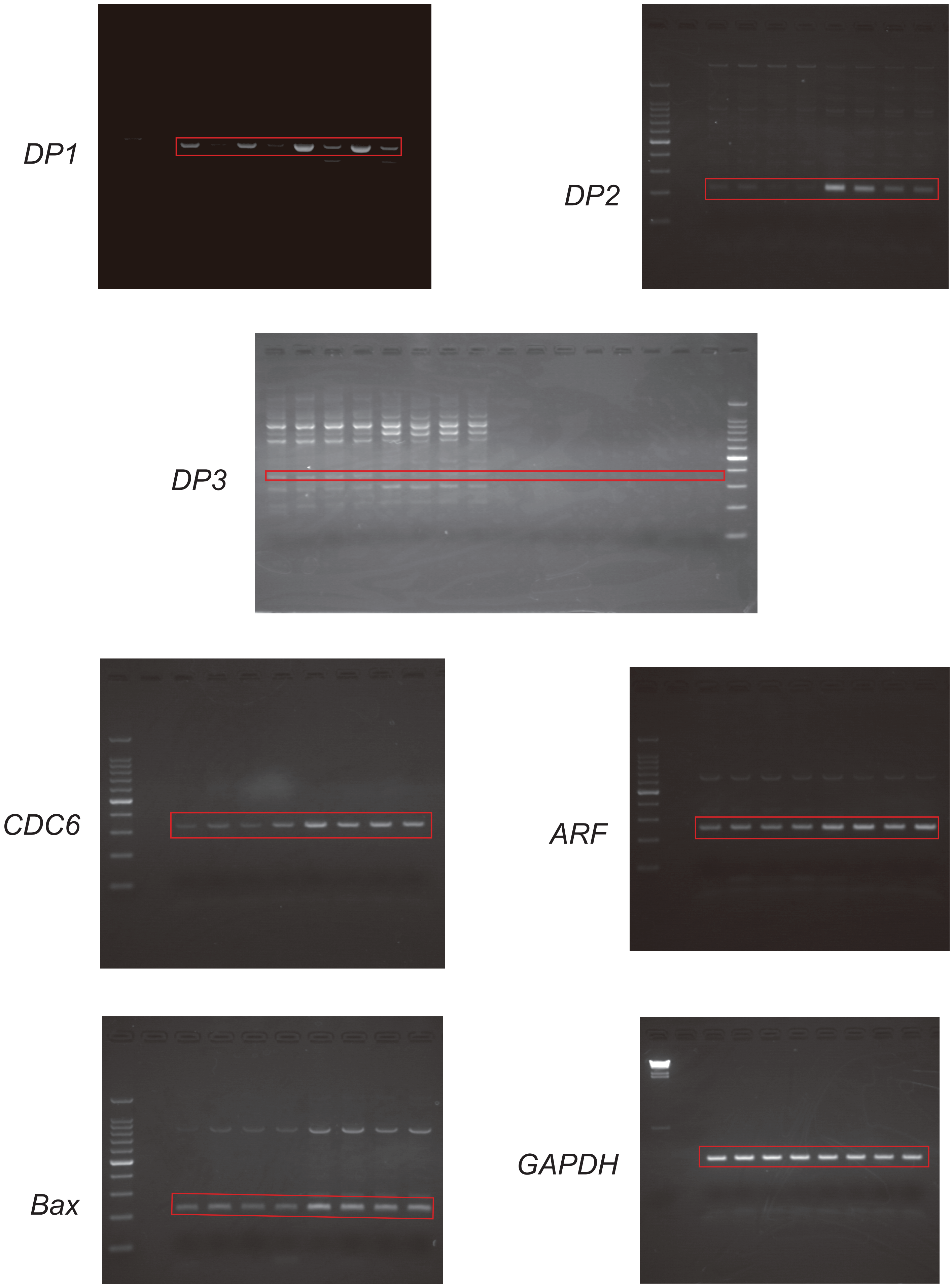

Supplementary Figure S15

Full-length gels of indicated PCR products illustrated in Figure 5(A).

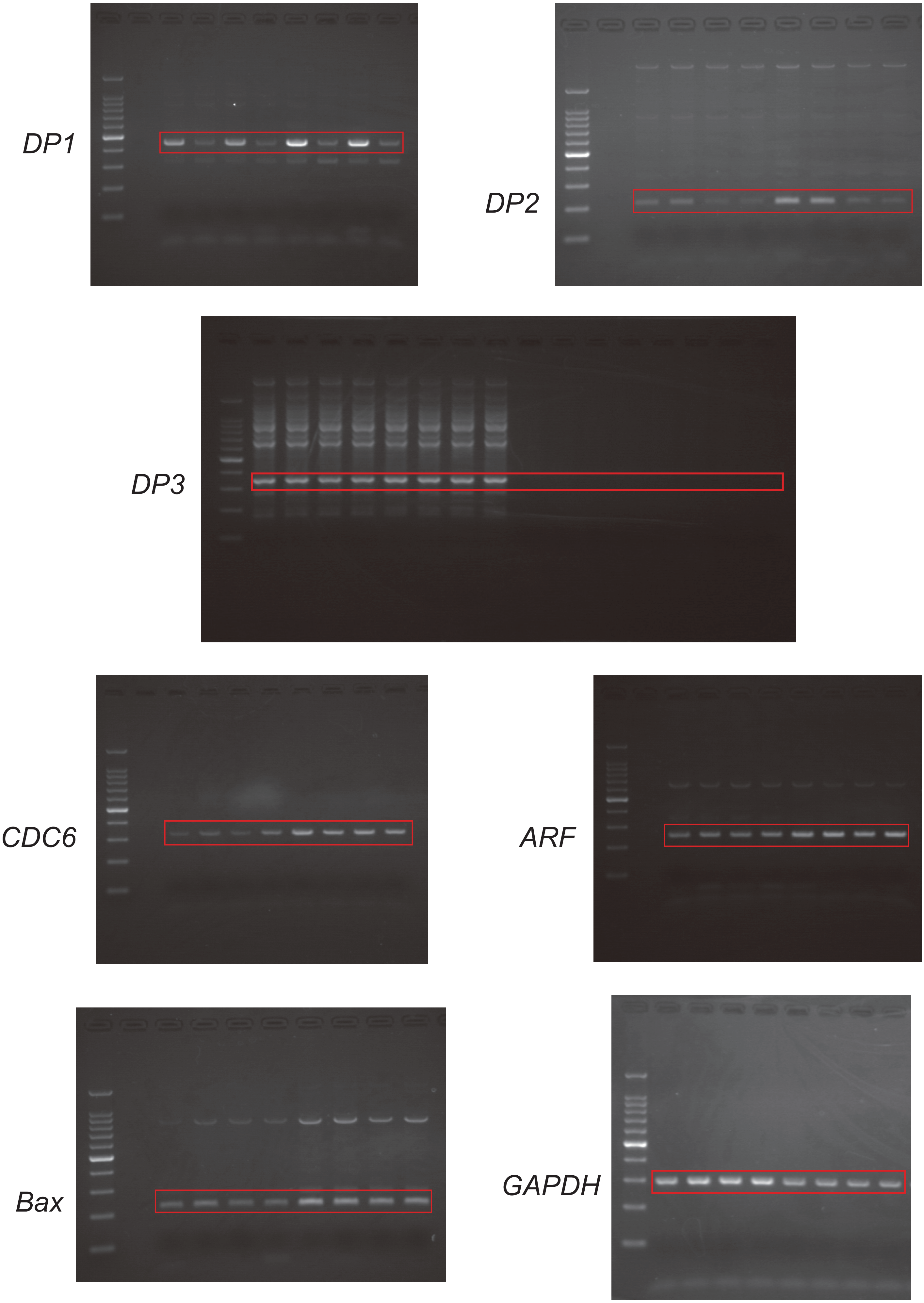

Supplementary Figure S16

Full-length gels of indicated PCR products illustrated in Figure 5(B).

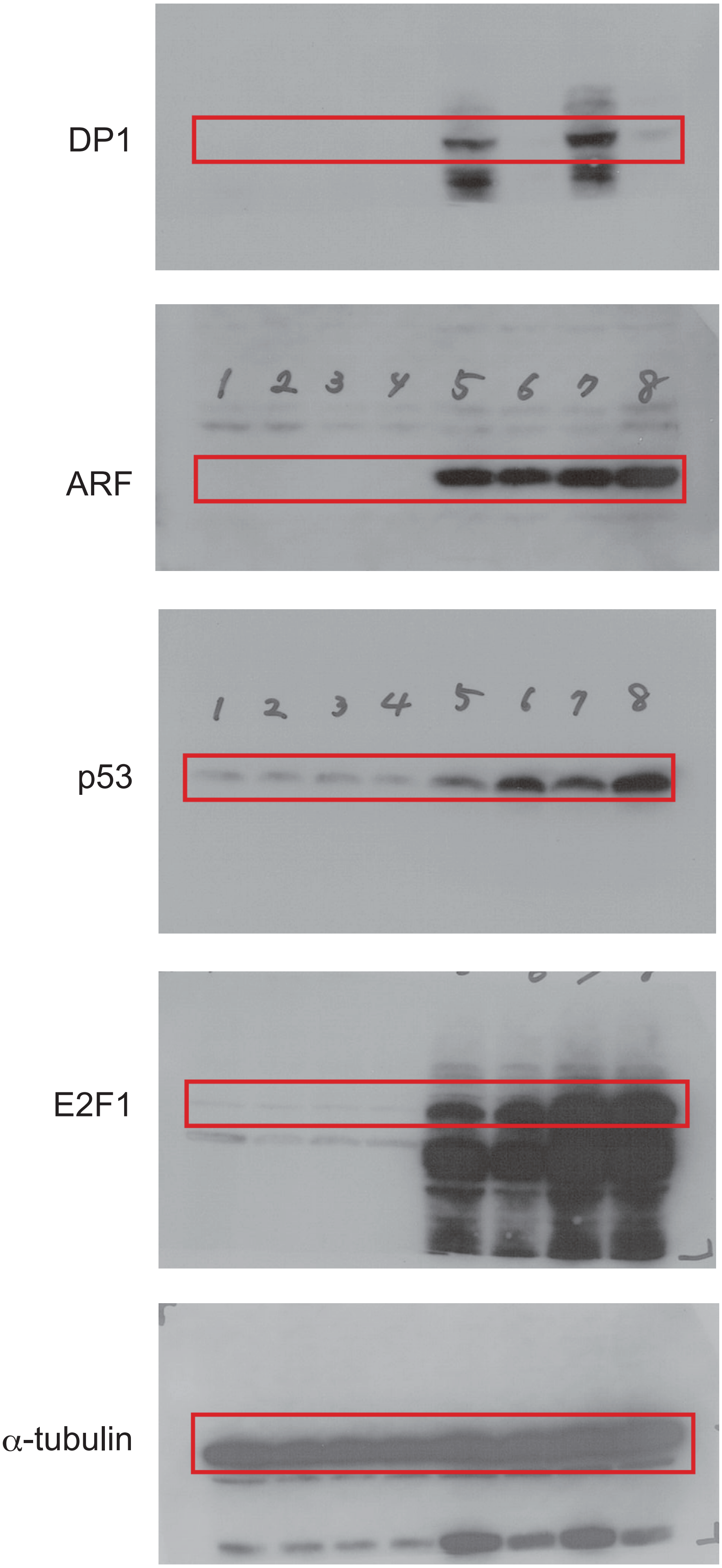

Supplementary Figure S17

Full-length blots of indicated proteins illustrated in Figure 5(C).

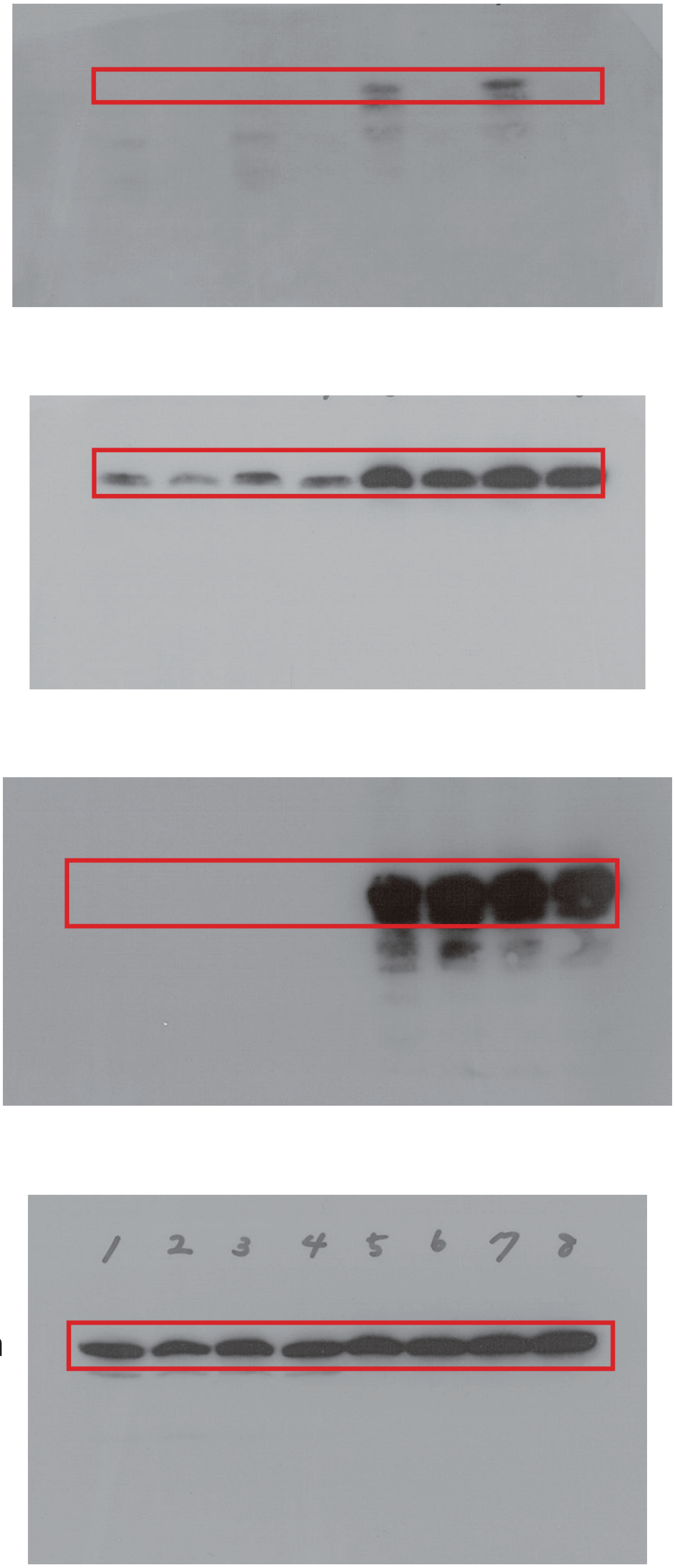

Supplementary Figure S18

Full-length blots of indicated proteins illustrated in Figure 5(D).

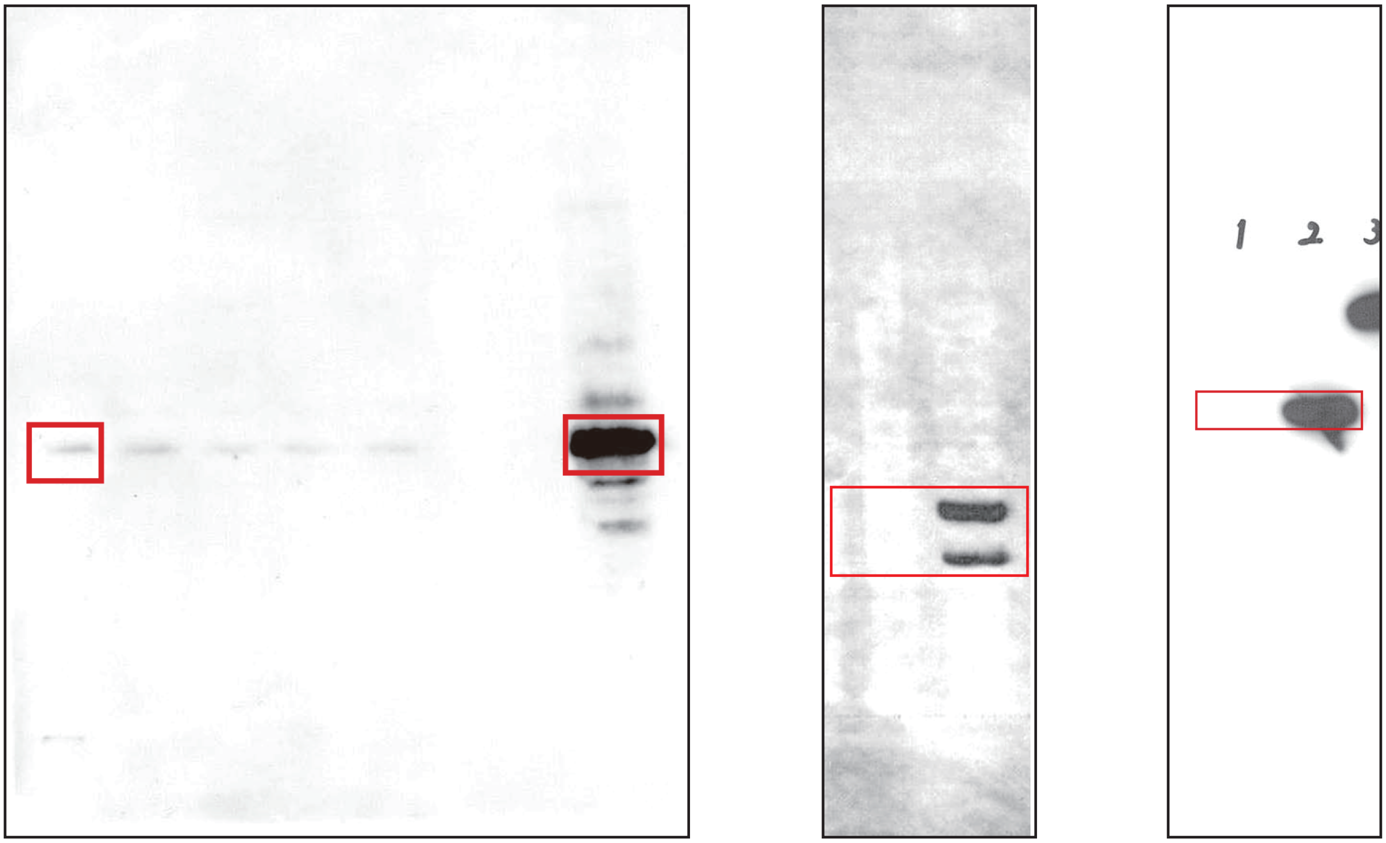

DP1 DP2 DP3

Supplementary Figure S19

Full-length blots of DP proteins illustrated in Figure 5(H).
